# Climate change and carnivores: shifts in the distribution and effectiveness of protected areas in the Amazon

**DOI:** 10.7717/peerj.15887

**Published:** 2023-09-19

**Authors:** Camila Ferreira Leão, Matheus S. Lima Ribeiro, Kauê Moraes, Gabriela Silva Ribeiro Gonçalves, Marcela Guimarães Moreira Lima

**Affiliations:** 1 Programa Pós-graduação em Ecologia, Universidade Federal do Pará, Belém, Pará, Brazil; 2 Laboratório de Biogeografia da Conservação e Macroecologia, Universidade Federal do Pará, Belém, Pará, Brazil; 3 Laboratório de Macroecologia, Universidade Federal de Goiás, Jataí, Goiás, Brazil; 4 Programa de Pós-graduação em Zoologia, Universidade Federal do Pará, Belém, Pará, Brazil

**Keywords:** Deforestation, SDM, Taxonomic richness, PA, Mammals, Carnivora

## Abstract

**Background:**

Carnivore mammals are animals vulnerable to human interference, such as climate change and deforestation. Their distribution and persistence are affected by such impacts, mainly in tropical regions such as the Amazon. Due to the importance of carnivores in the maintenance and functioning of the ecosystem, they are extremely important animals for conservation. We evaluated the impact of climate change on the geographic distribution of carnivores in the Amazon using Species Distribution Models (SDMs). Do we seek to answer the following questions: (1) What is the effect of climate change on the distribution of carnivores in the Amazon? (2) Will carnivore species lose or gain representation within the Protected Areas (PAs) of the Amazon in the future?

**Methods:**

We evaluated the distribution area of 16 species of carnivores mammals in the Amazon, based on two future climate scenarios (RCP 4.5 and RCP 8.5) for the year 2070. For the construction of the SDMs we used bioclimatic and vegetation cover variables (land type). Based on these models, we calculated the area loss and climate suitability of the species, as well as the effectiveness of the protected areas inserted in the Amazon. We estimated the effectiveness of PAs on the individual persistence of carnivores in the future, for this, we used the SDMs to perform the gap analysis. Finally, we analyze the effectiveness of PAs in protecting taxonomic richness in future scenarios.

**Results:**

The SDMs showed satisfactory predictive performance, with Jaccard values above 0.85 and AUC above 0.91 for all species. In the present and for the future climate scenarios, we observe a reduction of potencial distribution in both future scenarios (RCP4.5 and RCP8.5), where five species will be negatively affected by climate change in the RCP 4.5 future scenario and eight in the RCP 8.5 scenario. The remaining species stay stable in terms of total area. All species in the study showed a loss of climatic suitability. Some species lost almost all climatic suitability in the RCP 8.5 scenario. According to the GAP analysis, all species are protected within the PAs both in the current scenario and in both future climate scenarios. From the null models, we found that in all climate scenarios, the PAs are not efficient in protecting species richness.

## Introduction

Human-induced climate change has become a major threat to biodiversity (IPCC, 2018). These shifts in climate affect biodiversity in several ways, and they may cause changes in the geographic distribution and phenological dynamics (seasonality of activities) of species (Forrest, 2015), affecting their permanence in certain regions (Walther et al., 2002). In response to such adverse conditions, species tend to migrate in search of suitable climatic conditions to ensure their survival (Parmesan & Yohe, 2003; Root et al., 2003; Garcia et al., 2014; Pecl et al., 2017). Since species have different environmental tolerances and respond differently to climate change, this displacement to suitable environments will depend on the dispersal capacity of each species, which may result in changes in the composition and abundance of communities, as well as disturbing biotic interactions (*e.g.*, pollination, predation, seed dispersal), altering the structure and functioning of communities (Cahill et al., 2013; Blois et al., 2013; Silva et al., 2015).

Among the possible species affected by climate change, tropical species are among the most vulnerable because they have very specific ecological niches and climate tolerances close to physiological limits due to the more stable climate in this region (Ribeiro, Sales & Loyola, 2018). Studies predict that the tropics may experience extreme droughts and warming climatic conditions (Loarie et al., 2009; Garcia et al., 2014), in the Amazon these impacts have already been recorded, where the duration of the dry season and its intensity have increased, while rainfall became more intense during the rainy season, threatening the biodiversity of this biome, which represents one of the largest in the world (Gloor et al., 2015; Esquivel-Muelbert et al., 2019). Future climate scenarios project that annual precipitation in the Amazon will fall rapidly and substantially (Cochrane & Barber, 2009), which, combined with a temperature increase to a critical limit, could lead to large-scale savannization events, mainly from the south. and eastern Amazonia (Nobre et al., 2016). According to Schloss, Nuñez & Lawler (2012), in the Amazon, the speed of climate change is seven times greater than the dispersion of animals, and 40% of mammals in the entire Western Hemisphere will not be able to keep up with these changes. If these scenarios materialize, the intensity and speed of these changes will challenge the ability of species to adapt to new climatic conditions and to disperse to suitable areas (Parmesan, 2006).

Along with climate change, Amazonian species also constantly face habitat loss and fragmentation through deforestation for land use, an impact that culminates in the reduction or extinction of biodiversity, thus compromising ecosystem functioning (Hanski, 2011; Haddad et al., 2015). Deforestation also causes changes in the availability of resources and refuges, composition and distribution of species, especially species with a positive relationship to forest cover, leading them to other locations or restricting them (Vieira et al., 2008; Regolin et al., 2017). Furthermore, deforestation contributes to climate variations from the local to the global scale, mainly through CO_2_ emissions into the atmosphere, causing an increase in temperature and a drop in local precipitation, intensifying periods of drought (Werth & Avissar, 2002; Nobre et al., 2009a; Lawrence & Vandecar, 2015).

In this context, carnivores (Carnivora, Mammalia) are species vulnerable to anthropogenic interference and have already experienced substantial population declines and contraction of a considerable geographic range (Crooks et al., 2011; Ripple et al., 2014). Due to their specific biological characteristics, such as area and population requirements (small populations, low reproductive rates, and large home ranges), they could potentially negatively experience the effects of climate change (Crooks, 2002; Rich et al., 2017; Arias-Alzate et al., 2020). The main causes for this decline are related to the loss or degradation of their habitats and prey, retaliation by humans, as well as their large-scale exploitation for traditional medicine and sport hunting (Cardillo et al., 2004; Di Minin et al., 2016).

The loss of carnivore species can cause instability in the ecosystem, as they are key species for the regulation and functioning of communities (Schmitz, Hambäck & Beckerman, 2000; Di Minin et al., 2016). Top predators (large carnivores) limit the abundance and distribution of their prey and, consequently, the trophic levels below (control known as Top-down) so that their removal from the community would result in a trophic cascade, that is, lack of population control of all trophic levels below (Del Rio et al., 2001; Estes et al., 2011; Ripple et al., 2014). Mesopredators (small and medium-sized carnivores) are seed dispersers and also regulate prey through predation (Belant, Schipper & Conroy, 2009). In cases of a trophic cascade, mesopredators can reestablish the balance of the community with predation rates similar to top predators; however, they do not completely replace them in their functions, and the increase in their populations can result in an uncontrolled increase in predation (Prugh et al., 2009; Brook, Johnson & Ritchie, 2012; Di Minin et al., 2016). Hence, regardless of size, carnivores are fundamental in the networks of interactions, dynamics, and structure of communities, being key points for conservation (Ripple et al., 2014; Di Minin et al., 2016).

Considering all these factors and the dynamic state of climate change scenarios, there is a complex spatial problem for conservation since these processes can affect the persistence of species in areas that are currently destined for conservation (Protected Areas - PAs) (Araújo et al., 2004; Soares-Filho et al., 2010). In this way, PAs may become insufficient and/or inadequate since these protected areas are located in geographic areas that, during their proposal, did not take into account this dynamic state of climate change (Hannah et al., 2013; Lemes & Loyola, 2013). In addition, climate change poses major challenges for conservation planning since species distributions are affected in a complex and particular way (Hannah et al., 2013; Lemes & Loyola, 2013; Alagador, Cerdeira & Araújo, 2016).

Thus, knowledge about species distributions is fundamental for macroecology and conservation studies. One of the tools currently being used in the analysis of biodiversity conservation is Species Distribution Modelling (SDM) (Elith & Graham, 2009). SDMs predict the environmentally suitable areas for species by correlating environmental variables with occurrence records and mapping their potential geographic distribution in the present and future (Guisan & Zimmermann, 2000; Guisan & Thuiller, 2005; Peterson & Soberón, 2012). SDMs have been frequently used to assess the effects of climate change on the geographic distribution of organisms (Giannini et al., 2012; Miranda, Imperatriz-Fonseca & Giannini, 2019; Giannini et al., 2020).

Based on the SDMs, we evaluated the impact of climate change on the geographic distribution of carnivores in the Amazon. Do we seek to answer the following questions: (1) What is the effect of climate change on the distribution of carnivores in the Amazon? (2) Will carnivorous species lose or gain representation within the Protected Areas (PAs) of the Amazon in the future?

## Materials & Methods

### Study area

**Figure 1:**
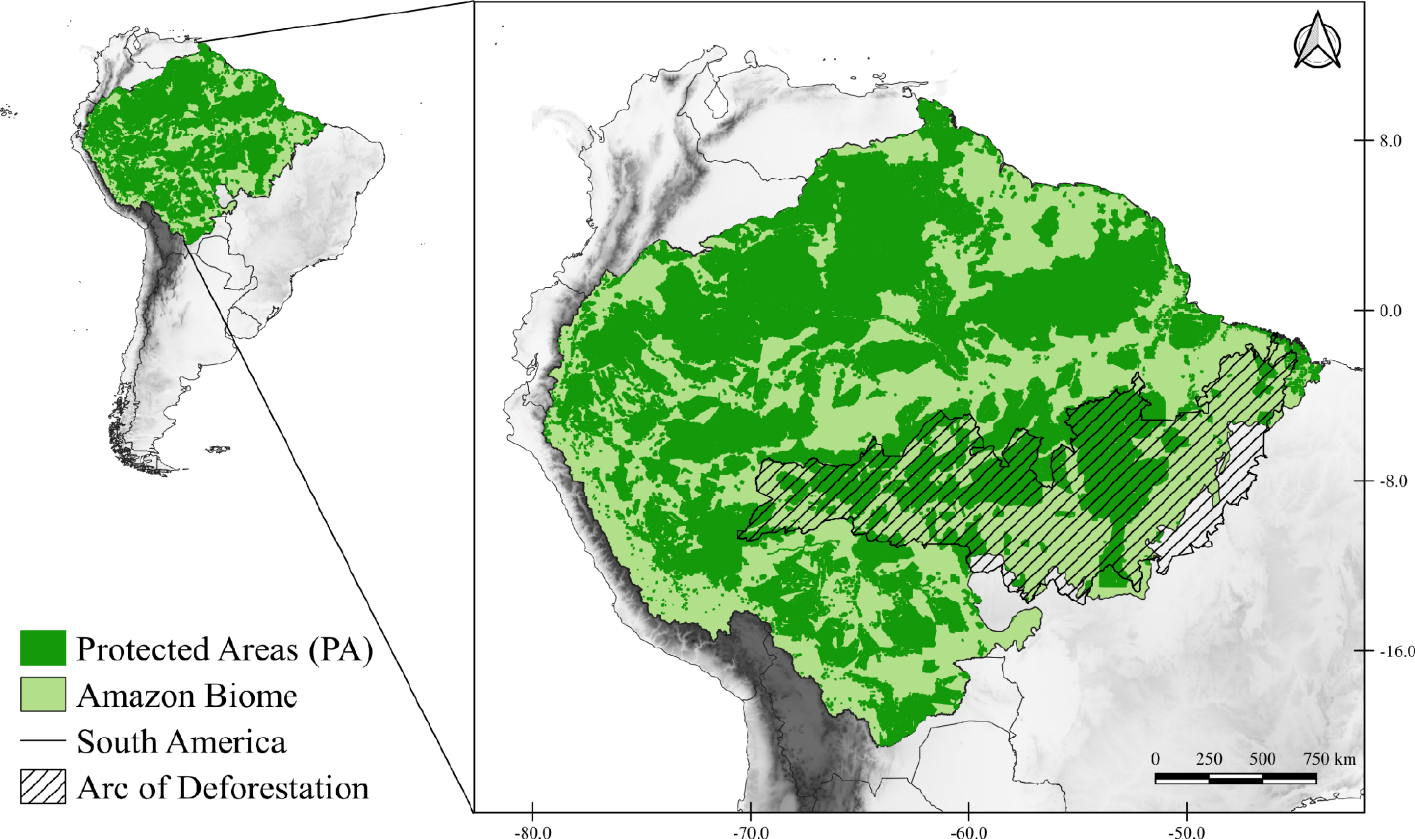
Map of the study area. Map of the Amazon Biome (solid light green color), corresponding to the study area. All protected areas (PA) of the Biome are represented on the map in solid dark green. The arc of deforestation is represented in striped form.

The study area corresponds to the Amazon Biome (Fig. 1), which covers nine South American countries (Brazil, Bolivia, Peru, Colombia, Venezuela, Suriname, Guyana, French Guiana, and Ecuador). The Brazilian territory contains most of the biome, with about 4,196,943 km^2^ (Ministerio do Meio Ambiente MMA, 2021). The biome has an average rainfall of 2,300 mm/year, reaching 5,000 mm/year in the western portion of the biome (Marengo & Nobre, 2009), and temperatures that vary between 24 °C and 26 °C, with amplitudes of 1 to 2 °C (Nobre et al., 2009b). The Amazon is very complex and heterogeneous that presents different landscape compositions (tropical forest, transitional forest, and tropical savanna), consisting of 94% humid forests, 4% flooded and 2% dry forests (Levine et al., 2015; DeArmond et al., 2023). Due to its wide diversity it is subdivided into areas of endemism, those areas of endemism are separated according to rivers, each area presents a set of unique species that are not present in other regions (Da Silva, Rylands & Fonseca, 2005).

### Target species, occurrence records and data cleaning

We obtained occurrence records for the 16 species of terrestrial carnivores belonging to the order Carnivora (Mammalia) residing in the Amazon Biome (Table S1) from online databases such as the Global Biodiversity Information Facility (http://www.gbif.org/) (Table S2), the Biodiversity Portal (https://portaldabiodiversidade.icmbio.gov.br/), Vertnet (http://www.vertnet.org/index.html) and speciesLink (http://splink.cria.org.br/). Furthermore, as a way to complement our database, records obtained from data papers (Lima et al., 2017; Grilo et al., 2018; Morato et al., 2018; Souza et al., 2019) and in primary and secondary literature (scientific articles, books, theses and published reports). To identify possible updates of scientific names and synonyms, we performed a taxonomic check of all target taxa. All records were georeferenced and geographic information of locations and municipalities whose coordinates were unavailable was obtained using Google Earth version 7.1.2 (https://www.google.com/earth/).

As a database cleaning procedure, we kept only occurrences identified at the species level. In addition, we discard data with unreliable geographic coordinates or approximate locations (for example, outside the species’ geographic range or that could not be confirmed). To correct geographic bias, we created grids with ∼20 km resolution (twice the resolution of the environmental variables) and then randomly selected only one occurrence record in each pixel (Velazco et al., 2019). We performed this procedure using the spThin package (Aiello-Lammens et al., 2015).

### Environmental data

For the current, we used the 19 bioclimatic variables obtained from the Worldclim 1.4 platform (http://worldclim.org) at a spatial resolution of 5 arc minutes (∼10 km). For the future scenario, we used the same 19 bioclimatic variables simulated from Atmosphere-Ocean General Circulation models (AOGCMs) for 2070. We performed a cluster analysis to select the AOGCMs that maximize the uncertainty among the climate models. We set an AOGCM from each group, as proposed by Varela, Lima-Ribeiro & Terribile (2015). Therefore, we selected five AOGCMs: CCSM4, HadGEM2-AO, IPSL-CM5A-LR, MRI-CGCM3, and MIROC-ESM. To assess the effect of climate on species, we considered two Representative Concentration Pathways (RCP) scenarios, RCP 4.5 (synonymous: RCP 45) as the mitigation scenario (optimist) and RCP 8.5 (synonymous: RCP 85) as the scenario without restrictions on gaseous emissions (pessimistic). We restricted the bioclimatic variables with the mask of the American continent, which corresponds to the total range of the distribution of the target species.

To avoid collinearity between the climatic variables, we performed a Principal Component Analysis (PCA) to reduce the dimensions of the bioclimatic variables (De Marco & Nóbrega, 2018). We used only the axes that explained at least 95% of the original variance of the 19 bioclimatic variables as predictors of the response functions. Hence, we selected the first six axes (Table S3). To maintain the dimensionality of climate data over time, we used the coefficients obtained from the PCA performed with present climate data to compute scores for future climate data for each selected AOGCM (Sillero & Barbosa, 2021).

### Species distribution models and taxonomic richness

Seeking to contemplate the uncertainty between algorithms, we use five methods commonly used in SDMs that stand out for their performance (Velazco et al., 2017), namely: Maximum Entropy (MaxEnt), support vector machine (SVM), random forest (RF), generalized linear model (GLM) and Gaussian process (GAU). MaxEnt is an algorithm based on presence background (Elith, Kearney & Phillips, 2010), which analyzes the actual occurrence points of species and relates them to the study area (background). The other four algorithms use presence-absence methods (Breiman, 2001; Tax & Duin, 2004; Golding & Purse, 2016). As there is no knowledge of absence points for the species studied, an environmental restriction method was used that randomizes points within climatically suitable areas for the species, thus creating pseudo-absences (Engler, Guisan & Rechsteiner, 2004), with a ratio of 1 point of pseudo-absence for each point of presence.

To improve the accuracy of the SDMs, we performed two statistical methods of partitioning the occurrence records: checkerboard and K-fold. For species with 15 or more occurrences, we used the geographically structured cross-validation method in checkerboard blocks to control the spatial autocorrelation between training and test data (Muscarella et al., 2014; Roberts et al., 2017). In this method, checkered grids have generated that partition the training and test data into blocks along the entire geographic extension, where it explores spatial blocks with different cell sizes so that the best cell resolution can be found for each species, being thus selecting the best answer for a given set of presence and presence-pseudo-absences (Velazco et al., 2019). In this way, the best resolution can be achieved by the simultaneous presentation of (a) the smallest spatial autocorrelation (measured by the Moran I index), (b) the maximum environmental similarity (measured by the Euclidean distance), (c) the minimum difference of records between the training and test data (Standard Deviation, SD) (Velazco et al., 2019), taking into account the transferability of models more directly, and providing more robust estimates in situations of studies with different time scales (Santini et al., 2021). We tested different cell resolutions ranging from twice the resolution of climate variables to 10 degrees, resulting in the optimal cell size for cross-validation (Velazco et al., 2019). We used the K-fold method for species with less than 15 occurrence records, which is adequate for few occurrence records (Fielding & Bell, 1997). This method divides the dataset into K random folders, where the models are adjusted into K-1 parts for training and the rest of the data for testing (Jung & Hu, 2015; Andrade, Velazco & De Marco Júnior, 2020). For our models, we used three random folders.

The predictive performance of the models was evaluated using the Jaccard similarity index (Jaccard, 1908) and area under curve (AUC) (Lobo, Jiménez-Valverde & Real, 2008). The Jaccard index measures the similarity between predictions and observations so that the closer the value is to 1, the greater the correspondence between both, and the smaller the number of false positives and negatives (Leroy et al., 2018). The predictive performance AUC method, calculated from the receiver operating characteristic (ROC) curve, is independent of decision thresholds and uses binary data to make predictions (Lobo, Jiménez-Valverde & Real, 2008). These data develop a two-by-two matrix with four elements: sensitivity, specificity, commission, and omission errors, and then model validation values are generated (Lobo, Jiménez-Valverde & Real, 2008). AUC values less than or equal to 0.5 indicate random predictions, while AUCs equal to 1 shows perfect predictions (Lima-Ribeiro & Diniz-Filho, 2012). However, AUC values above 0.75 are considered helpful for model evaluation (Lima-Ribeiro & Diniz-Filho, 2012).

Finally, we used an ensemble forecasting approach (Araújo & New, 2007; Lima-Ribeiro & Diniz-Filho, 2012) obtained through a simple average of the algorithms and only the algorithms above this average are selected for the ensemble.. We did this process for the present and each of the future climate scenarios (RCP 4.5 and 8.5), generating the consensus models. To avoid models with erroneous or/and highly extrapolated distributions, all present consensus models were compared with the actual distribution of the species (available on the IUCN platform) and were evaluated by experts. All modeling and model evaluation procedures were performed using the ENMTML package (by Andrade, Velazco & De Marco Júnior, 2020) using R software v4.0.3 (R Core Team, 2022).

### Overlap of species distribution models with vegetation cover models

We converted the continuous climate prediction models into binary presence and absence models, employing a threshold with the values that maximize the specific Jaccard for each species. Subsequently, as carnivorous mammals include species that are generally negatively affected by landscape changes (Regolin et al., 2017), we overlap the climate models with a vegetation cover model (land type) proposed by Chen, Li & Liu (2022) for all target species. We use a different vegetation cover model according to each climate scenario (three models). For this, we selected the types of vegetation cover that have the most significant influence on the distribution of the studied species, namely: Broadleaf evergreen tree, tropical; Broadleaf deciduous tree, tropical; Broadleaf deciduous tree, temperate; Broadleaf deciduous tree, boreal; Broadleaf deciduous shrub, temperate.

### Post processing

To verify the areas with the greatest species richness, we produced richness maps for the present and each future climate scenario (optimistic and pessimistic). The maps were prepared from the sum of all the final models of each species, using the raster calculator tool in the Qgis 3.16 (Q GIS Development Team, 2020).

We then calculated the size of potential distribution for each species for the current and future scenarios using the raster package (Hijmans, 2020) implemented in R software v4.1.3 (R Core Team, 2022). Considering each climatic scenario, we calculated the loss/gain of a potential distribution based on the difference between the present and the future species distribution area. For the taxon *Atelocynus microtis*, we used a geographic restriction mask to correct the extrapolation of the current distribution generated by the model; this procedure was done with the aid of the study by Rocha et al. (2020) using Qgis 3.16.

### Effectiveness of protected areas

To represent the PAs located in the Amazon Biome (Fig. 1), we consider three categories of PAs, namely: (1) Integral Protected Areas (IPA), (2) Sustainable Use Areas (SUA), and (3) Indigenous Lands (IL) obtained through a compilation among the databases of the World Database on Protected Areas (WDPA) (https://www.protectedplanet.net/, UNEP-WCMC; IUCN, 2022), Amazonian Network of Georeferenced Socio-Environmental Information (https://www.raisg.org/) and the Ministry of the Environment (http://mapas.mma.gov.br/i3geo/datadownload.htm). Seeking to maintain consistency with the spatial scale of the SDMs (∼10 km) and not to overestimate the number of species in PAs with small sizes, PAs smaller than 50 km^2^ were excluded from the analyzes (Velazco et al., 2022). In addition, we only consider the part included within the Amazon Biome for PAs that cover marine territories or exceed the study area. Thus, the total number of PAs selected was 2,565, comprising 45,578 cells distributed over 3,908.313 km^2^ of the Amazon Biome. We performed all analyzes cited in this topic for all climate scenarios using R software v4.0.3 (R Core Team, 2022).

We identified the effectiveness of PAs in protecting each carnivores through Gap Analysis, proposed by Rodrigues et al. (2004). This analysis relates the species distribution with the distribution of PAs in the Amazon according to a target, generating representativeness levels within the PAs for each species. The gap analysis proposes that species with a restricted distribution, with a distribution area smaller than 1,000 km^2^, should have 100% of their distribution protected. In comparison, species with a distribution area greater than 250,000 km^2^ should have at least 10% of their area protected. For species with intermediate distribution, the calculation is performed through interpolation using a logarithmic transformation, following the methods proposed by Rodrigues et al. (2004).

In this way, we classify each species according to the degree of the conservation goal reached, according to Frederico, Zuanon & De Marco (2018), as (1) Protected (P) when ≥90% of the target percentage of the species distribution size is within of PAs; (2) Partially Protected (PP) when <90% and ≥70% of the target is within the PAs; (3) Gaps (G) when <70% and ≥20% of the target is within the PAs, and; (4) Not Protected (NP) when a very small part (<20%) of the target percentage was within the PAs. We used the null model approach to assess the effectiveness of PAs in protecting all species together (taxonomic richness) in the present and future scenarios (Ribeiro et al., 2016; Velazco et al., 2021). This approach indicates the ability of PAs to retain species richness different from what is expected by chance. For this, each PA was randomized 999 times throughout the Amazon, maintaining the PA’s size, orientation, and shape, where the richness values are calculated in each randomization within the protected cells. Suppose the observed richness values are at least 95% above the values expected by chance (*p* < 0.05), the PA is classified as effective in protecting the richness. Subsequently, we calculated each climate scenario’s total proportion of protected richness in PAs. Data were analyzed using raster and dplyr packages through R software v4.0.3 (R Core Team, 2022).

## Results

A total of 27,033 records of unique occurrences were obtained for all 16 species of terrestrial carnivores (Carnivora, Mammalia) from the Amazon, with *Mustela africana* having the lowest number of occurrences (14) and *Puma concolor* with the highest number of occurrences (4,652) (Table S4). The SDMs showed satisfactory predictive performance, with Jaccard values above 0.85 and AUC above 0.91 for all species (Tables S4 ; S5).

**Figure 2:**
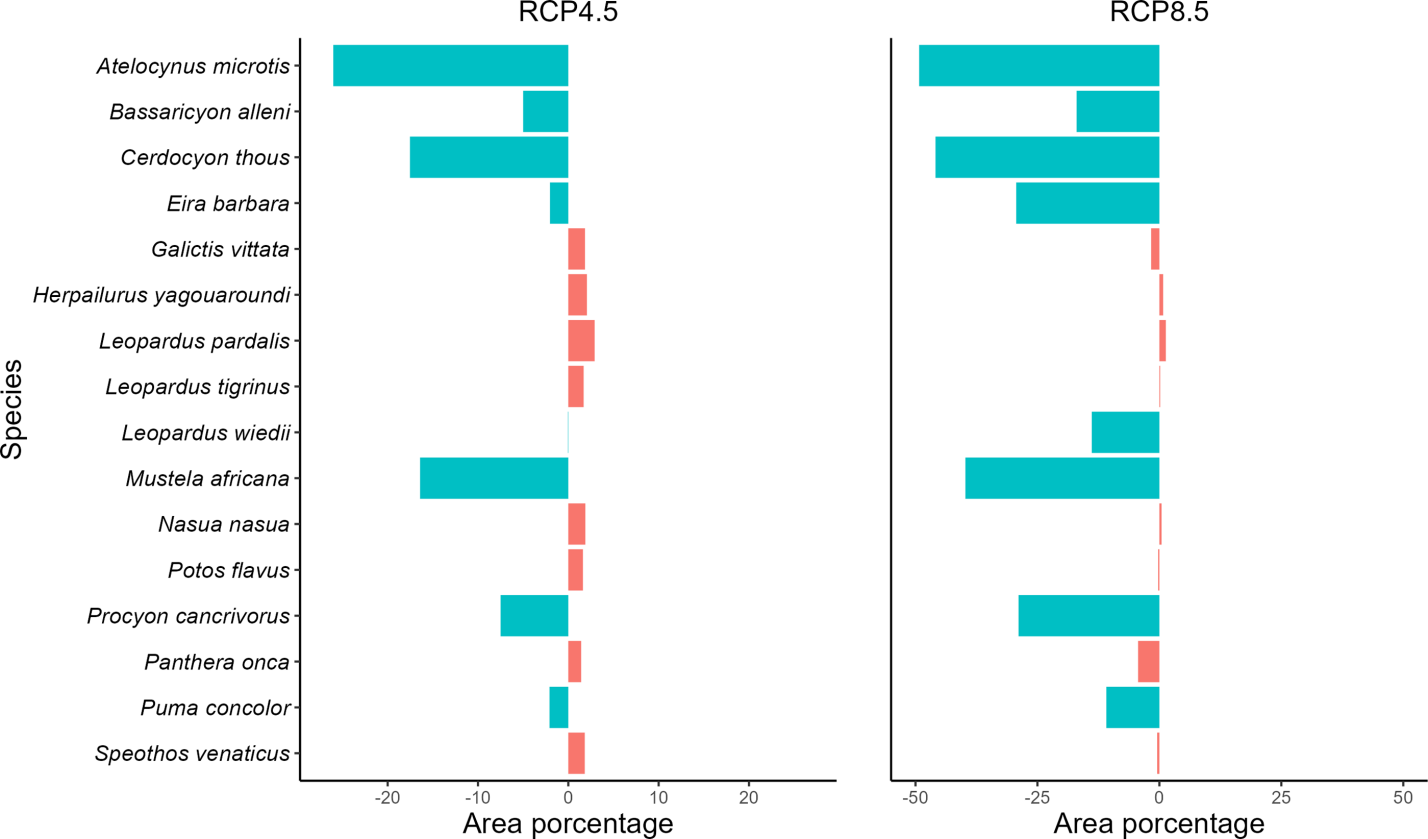
Measuring spatial dynamics. Values in percent of potential distribution loss or gain by carnivore species. Graph in percent of the loss or gain of the potential distribution of each carnivore species. Values with the negative sign represent loss of spatial distribution and positive values represent gains. Species with loss/gain of potential distribution less than 5% are considered stable. More details can be found in Table S6.

Analyzing the SDMs of the taxa in the present and for the future climate scenarios, we observe a reduction of potencial distribution in both future scenarios (RCP4.5 and RCP8.5), where five species will be negatively affected by climate change in the optimistic future scenario and eight in the pessimistic scenario (Fig. 2, Fig. S1 and Table S6). On the other hand, 11 species in the optimistic and eight in the pessimistic scenario may remain stable, with gains or losses in potencial distribution of less than 5%. They will retain their potencial distributions if the projections materialize. For both scenarios, no species showed a significant gain in the area.

Regarding the optimistic scenario (RCP 4.5), the species exhibit a loss of up to 26% of the potencial distribution, with the lowest loss of 5% for the species *Bassaricyon alleni* and the highest loss of 26.02% for the species *A. microtis*. In addition, the taxa *Cerdocyon thous*, *M. africana*, and *Procyon cancrivorus* are also negatively affected by climate change, losing, respectively, 17.53%, 16.41%, and 7.5% (Fig. 2 and Table S6). For the pessimistic climate scenario (RCP 8.5), the area losses in relation to the optimistic scenario and the present one were more substantial, with the smallest area loss registered for *P. concolor* (10.88%), which previously presented stability, and the greatest loss was 49.25% for *A. microtis*. The carnivores *B. alleni*, *C. thous*, *Eira Barbara*, *Leopardus wiedii*, *M. africana*, and *P. cancrivorus* also lost potencial distribution in the pessimistic scenario (Fig. 2 and Table S6). The remaining species remained stable in this scenario (Fig. 2 and Table S6).

All species in the current study showed a loss of climatic suitability, including the species that remained stable in potencial distribution (Fig. S2). Most carnivores lost up to half of their climate suitability in the RCP 4.5 climate scenario, corresponding to five species that lost area in the future and nine stable species. In the RCP 8.5 climate scenario, four species also lost up to half of their climatic suitability, with three losing areas and two stables. In the RCP 8.5 climate scenario, most species lost more than half of their climate suitability, with five species also losing area and four classified as stable. We highlight the species *P. concolor*, classified as stable in the RCP 4.5 climate scenario, which lost more than half of its climate suitability in this scenario; in addition, it lost almost all its suitability in the pessimistic scenario.

**Figure 3:**
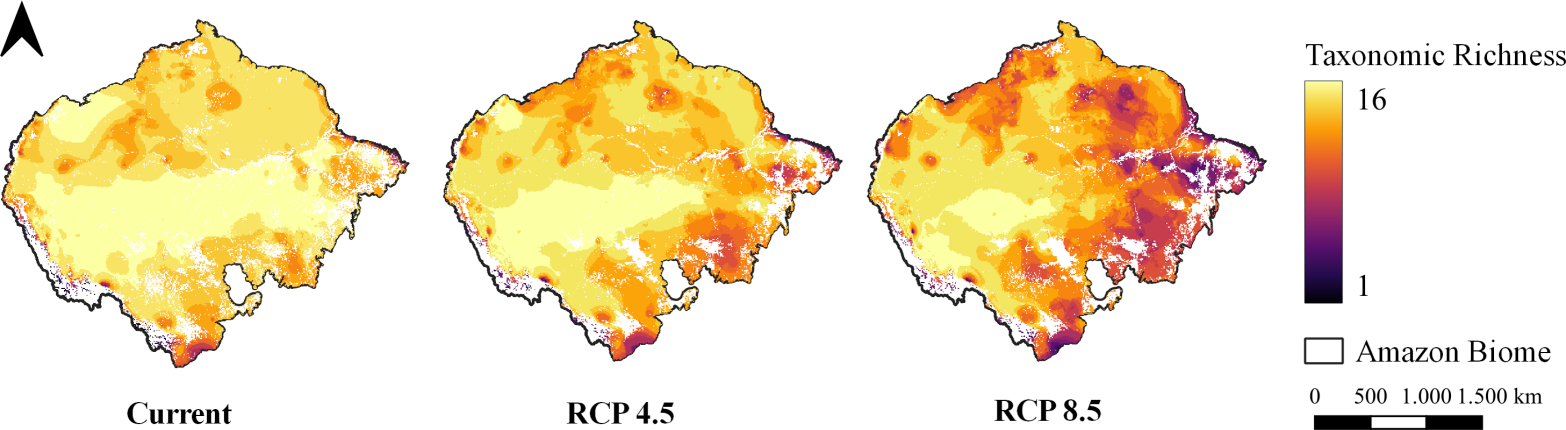
Taxonomic richness of target carnivores. Map of taxonomic species richness under all climate scenarios. Maps were generated from the overlapping SDMs (with forest remnant) of all species for each climate scenario, totaling 16 levels. Richness ranges from one species to 16 species together at the same site.

The pattern of species richness observed in the present indicates high richness uniformly distributed throughout the entire Amazon (Fig. 3). In the optimistic climate scenario, it is possible to observe a shift in the richness pattern towards the western region of the Biome (Fig. 3). In the pessimistic climate scenario, however, it is possible to observe a reduction in richness in the eastern region of the Amazon and almost the entirety of the deforestation arc (Fig. 3).

According to the GAP analysis, all species are individually protected within the PAs (IPA, SUA and IL) both in the current scenario and in both future climate scenarios, where all species have reached the conservation goal established by the analysis (Table S7). Analyzing the null models and species as a group, we found that in all climate scenarios, the PAs are not efficient in protecting species richness; that is, the ability to retain richness is not different from what is expected by chance (Fig. S3).

## Discussion

Our results demonstrate that climate change will affect the future distribution of carnivore mammals in the Amazon (2070) by reducing their potencial distributions and suitable climate. In the optimistic climate scenario, the species will have area losses of up to 26% compared to the present, and in the pessimistic scenario, up to 49%. Species will be represented individually in PAs in both climate scenarios. However, the taxonomic richness of carnivores will not be protected in either climate scenario. Some recent studies on the effects of climate change on carnivores corroborate our results, evidencing that this group will experience substantial loss of habitat and abundance, which may even cause its extinction (Barnosky et al., 2011; Visconti et al., 2016; Baisero et al., 2020). This pattern also seen for other taxonomic groups: birds (Jetz, Wilcove & Dobson, 2007), bats (Aguiar et al., 2016), felines (Arias-Alzate et al., 2017), plants (Velazco et al., 2019) and mammals in general (Ribeiro, Sales & Loyola, 2018).

In both climate scenarios, the species *A. microtis*, an endemic and threatened canid from the Amazon (Rocha et al., 2020), will be significantly affected by climate change. This species mainly inhabits the deforestation arc and depends on continuous and preserved forest areas, so its main threats are climate change and habitat loss due to forest degradation (Michalski, 2010; Rocha et al., 2020). *A. microtis* occurs at low population densities and has already had considerable losses in its population and is currently in decline (Leite-pitman & Beisiegel, 2011). In our study, this species could lose about 49% of its distribution, a large part located in the arc of deforestation, in addition to observing a significant loss of climate suitability for the future, mainly in the pessimistic scenario. However, the remaining part is also concentrated in the arc, endangering the persistence of the species. Our results also corroborate what Oliveira et al. (2022), which showed the loss of area for *A. microtis*, *C. thous*, and *Speothos venaticus* as a consequence of climate change for the year 2050. According to our projections, this scenario will probably continue and may lead to the formation of isolated populations and unfeasible (Leite-pitman & Beisiegel, 2013; Rocha et al., 2020). We emphasize the need for conservation measures for this species, which, in addition to being endemic, has several threats that endanger its future.

Our results demonstrate that the two big cats of the Amazon biome, *P. concolor*, and *Panthera onca*, will not significantly reduce the area in future scenarios. This apparent stability also corresponds to past times during the Quaternary climatic oscillations (Arias-Alzate et al., 2017). Despite this low loss of potencial distribution for these species, both present a considerable reduction in their climatic suitability in the future, compromising their survival. It is worth noting that most big cats are solitary and maintain little overlapping intrasexual home ranges, so the slightest contraction of their areas can lead to population reductions, increasing the vulnerability of these species due to the demographic bottleneck (Agosta & Bernardo, 2013), which together with other stressors such as loss of prey (which may suffer area reductions), hunting and habitat fragmentation (Zanin, Palomares & Brito, 2014) can operate synergistically.

Although some threatened species do not have their distribution negatively affected by climate change, as they remain stable in the future, we observed that all showed a large reduction in climate suitability in both future scenarios, mainly in the pessimistic scenario. The loss of suitability indicates climate change’s effects on the species (Leão et al., 2021), which may substantially increase their risk of extinction (Moat, Gole & Davis, 2019). For mammals, we also have the same projection of a loss of climate suitability for South America, where half of its species may lose 20% of suitability and a quarter may lose more than 50% (Baisero et al., 2020). In addition, there are several threats to carnivores that, together with the lack of climate suitability and climate change, can cause disturbances and the extinction of these species, such as, for example, the predatory hunting of these animals and their prey and the constant loss of habitat due to deforestation. Illegal (MacDonald, 2016; Farris et al., 2017).

Among the species that do not have any degree of threat, five (*B. alleni*, *C. thous*, *E. barbara*, *M. africana*, and *P. cancrivorus*) will be affected with reductions in the suitable climate area of up to approximately 46%. These small-sized carnivores, known as mesopredators, have been recognized as global sentinels of ecosystem structure, function, and change (Marneweck et al., 2022). They are omnivorous seed dispersers, where in the absence of the top predator, they take their place in the food chain (Prugh et al., 2009; Rather, Kumar & Khan, 2020). In addition, *C. thous* and *M. africana* may have their status compromised due to losing at least 30% of their geographic range (Purvis et al., 2000; Cardillo et al., 2005; International Union for Conservation of Nature, 2018). If these species are included in conservation plans, the impacts may be mitigated in the long term, and extinction or change of status will not occur for them (Soares-Filho et al., 2010).

Historically, deforestation and climate change are related to extreme drought events in the Amazon. This variation in the drought regime affects biodiversity, as we can observe in the shift in the richness of the present study (Esquivel-Muelbert et al., 2019; Staal et al., 2020). Deforestation and forest degradation, due to direct human intervention or droughts, reduce evapotranspiration, and therefore moisture is transported further westwards, reducing rainfall and forest viability in the rest of Amazonia, suggesting that vegetation in stressed regions of more pronounced aridity is at greater risk of losing resilience (Boulton, Lenton & Boers, 2022). As noted, species richness will be lost in the northern and eastern parts of the Amazon, coinciding with the regions that have experienced more extreme droughts in recent years and concentrating in the western, wetter portion (Malhi et al., 2008; Cox et al., 2008).

In short, the pattern of richness observed for the future (2070) in both climate scenarios is concentrated in western Amazonia, a region less impacted by deforestation and anthropic actions in general, and which therefore has the lowest future projections of deforestation rates (Vieira et al., 2008; Matavelia et al., 2021). Despite the low deforestation rates for this area compared to other regions, there is a concern with the future of this region, given that there is the implementation of road projects for this area. One of these projects is the paving of the BR-319 highway, which connects the capitals of Amazonas and Rondônia, crossing a site with 63 indigenous lands and several other Conservation Units (Ferrante, Gomes & Fearnside, 2020; Vilela et al., 2020; Matavelia et al., 2021).

This paving project would link the arc of deforestation with the central Amazon by opening up the large block of intact forest in the western portion of the Amazon, (Fernside & Graça, 2006; Ferrante, Gomes & Fearnside, 2020). Consequently, this project becomes worrying since the expected deforestation area should be about 170,000 km^2^ by 2050, and the forecast is to quadruple CO_2_ emissions (Soares-Filho et al., 2010). The concern regarding the implementation of this highway becomes evident when we see our richness map concentrated in the western region of the Amazon.

A substantial part of the richness that could be lost is located in the deforestation arc. In this region, the highest deforestation rates occur due agricultural development (Costa & Pires, 2010; Rehm et al., 2015).

Deforestation is driven by logging, mining, and especially road construction. Mining is currently one of the most worrying threats in Brazil, with plans to increase the number of projects by 10 times in 8 years with 11,000 proposed mining projects within PAs (Barni, Fearnside & Graça, 2015). In addition, there are also the impacts not accounted for by illegal mining, which represents 90% of artisanal mines in Brazil, may lead to significant forest losses extending up to 70 km from the mining boundaries (Sonter et al., 2017). This would directly affect the PAs, that be vital and indispensable for the conservation of species, acting to protect local communities and reduce population declines (Geldmann et al., 2013; Gray et al., 2016) and, consequently, the carnivores studied here.

Associated with good management, PAs may reduce human pressures (Kauano, Silva & Michalski, 2017). However, studies have already shown that PAs are disproportionately distributed in economically marginal lands to lower costs than in areas with greater biodiversity (Venter et al., 2018; Chen et al., 2022). Our results confirm this pattern, demonstrating that PAs cannot protect the richness of Amazonian carnivores in their current arrangement. However, when we analyze the species individually, it is possible to conclude that the PAs play a fundamental role in the conservation of carnivorous mammals in the Amazon because, despite the significant loss of suitable climatic areas for the species, all of them reach their protection goals both in the present as in future scenarios.

Nevertheless, despite PAs being an effective tool to reduce the impacts of land use disturbances, recent evidence indicates that many PAs still experience considerable human impact (Jones et al., 2018; Chen et al., 2022). Carnivores have large home ranges, and therefore they may frequent landscapes outside the PAs and co-occur with humans; even so, it is expected that the PAs reduce the anthropic pressures on these animals (Carter & Linell, 2016; Terraube et al., 2020). Although, biodiversity within PAs is declining due to human activities, and their existence alone is not enough to protect species (Kauano, Silva & Michalski, 2017). Deforestation is a constant threat within PAs; as a result, the representation rate of carnivores in PAs will reduce in the future, and the goal of protecting these animals through land use will be inadequate (Di Minin et al., 2016). Another threat to the persistence of carnivores is illegal hunting within PAs, including those with restricted use, being more intense in underdeveloped countries and large mammals (Rija et al., 2020). In addition to these problems, PAs are at serious risk of having their legal status lowered due to economic pressures on natural resources (Bernard, Penna & Araujo, 2014; Pack et al., 2016).

## Conclusions

Amazonian carnivores will be affected by climate changes in the future, according to our study. Reductions in the distribution and climatic suitability of these species will test their persistence in relation to human pressures, which may make these animals more vulnerable to stressors. Given this context, PAs have shown a fundamental role in the conservation of each carnivore, even with the negative effects of climate change. However, the conservation of these animals as a group will not be effective. Therefore, we signal that improvements are needed in the management and maintenance of the PAs, in order to make possible the total conservation of this relevant group. We point out that this study was carried out with environmental variables, and after building the model we included vegetation cover variables. Therefore, we do not take into account the dispersion of species, which can be a determining factor for the survival of carnivores in the face of climate change. Therefore, in the future, we intend to improve our studies by including dispersion variables and vegetation cover in the construction of SDMs. Even so, our SDMs showed important and decisive results for conservation and management decisions.

##  Supplemental Information

10.7717/peerj.15887/supp-1 Supplemental Information 1 Supplemental Material

10.7717/peerj.15887/supp-2 Supplemental Information 2 Database Records of occurrences (coordinates) of the target species.

## Additional Information and Declarations

### Data Availability

The following information was supplied regarding data availability: Raw data are available in the Supplemental Files.
