# Supplementary material for: Climate change and carnivores: shifts in the distribution and effectiveness of protected areas in the Amazon
Source: PeerJ. 2023 Sep 19;11:e15887. doi: 10.7717/peerj.15887 (PMC10516102; doi:10.7717/peerj.15887)
Supplement: Supplemental Information 1 [file peerj-11-15887-s001.docx]

**Table S1: List of mammals carnivorous species and and their threat status**. IUCN represents the global threat status and MMA the national threat status (From Brazil). Subtitles: LC (Least Concern); NT (Near Threatened); VU (Vulnerable); EN (Endangered); DD (Deficient Data). *Species with some threatened status.

| **Species** | **IUCN** | **MMA** |
| --- | --- | --- |
| **Canidae** | | |
| *Atelocynus microtis** | NT | VU |
| *Cerdocyon thous* | LC | LC |
| *Speothos venaticus** | NT | VU |
| **Felidae** | | |
| *Herpailurus yagouaroundi** | LC | VU |
| *Leopardus pardalis* | LC | LC |
| *Leopardus tigrinus** | VU | EM |
| *Leopardus wiedii** | NT | VU |
| *Panthera onca** | NT | VU |
| *Puma concolor* | LC | LC |
| **Procyonidae** | | |
| *Bassaricyon alleni* | LC | LC |
| *Nasua nasua* | LC | LC |
| *Potos flavus* | LC | LC |
| *Procyon cancrivorus* | LC | LC |
| **Mustelidae** | | |
| *Eira barbara* | LC | LC |
| *Galictis vittata* | LC | LC |
| *Mustela africana* | LC | DD |

**Table S2:** **Information about online database Gbif (Global Biodiversity Information Facility).** From left to right: carnivorous species, date of access to occurrence records for each species and DOI electronic address.

| Species | Access date | DOI |
| --- | --- | --- |
| *A. microtis* | 11 July 2020 | https://doi.org/10.15468/dl.m5fcqx |
| *B.alleni* | 11 July 2020 | <https://www.gbif.org/occurrence/download/0019456-200613084148143> |
| *C. thous* | 11 July 2020 | <https://www.gbif.org/occurrence/download/0019480-200613084148143> |
| *E. barbara* | 17 July 2020 | <https://www.gbif.org/occurrence/download/0023158-200613084148143> |
| *G. vittata* | 13 July 2020 | <https://www.gbif.org/occurrence/download/0020826-200613084148143> |
| *H. yagouaroundi* | 16 July 2020 | <https://www.gbif.org/occurrence/download/0023074-200613084148143> |
| *L. pardalis* | 13 July 2020 | <https://www.gbif.org/occurrence/download/0020859-200613084148143> |
| *L. tigrinus* | 13 July 2020 | <https://www.gbif.org/occurrence/download/0020653-200613084148143> |
| *L. wiedii* | 15 July 2020 | <https://www.gbif.org/occurrence/download/0022520-200613084148143> |
| *M. africana* | 13 July 2020 | <https://www.gbif.org/occurrence/download/0020732-200613084148143> |
| *N. nasua* | 22 July 2020 | <https://www.gbif.org/occurrence/download/0026273-200613084148143> |
| *P. cancrivorus* | 15 July 2020 | <https://www.gbif.org/occurrence/download/0022441-200613084148143> |
| *P. concolor* | 21 July 2020 | <https://www.gbif.org/occurrence/download/0026106-200613084148143> |
| *P. flavus* | 23 July 2020 | <https://www.gbif.org/occurrence/download/0027288-200613084148143> |
| *P. onca* | 18 July 2020 | <https://www.gbif.org/occurrence/download/0024444-200613084148143> |
| *S. venaticus* | 12 July 2020 | <https://www.gbif.org/occurrence/download/0019955-200613084148143> |

**Table S3:** **Results of Principal Component Analysis (PCA).** The six axes selected from the PCA that explained at least 95% of the original variance.

| PCA Axes | Variance - Principal Component | Cumulative Variance |
| --- | --- | --- |
| PC1 | 0.54622 | 0.54622 |
| PC2 | 0.20108 | 0.7473 |
| PC3 | 0.09501 | 0.84231 |
| PC4 | 0.05651 | 0.89882 |
| PC5 | 0.04112 | 0.93994 |
| PC6 | 0.02827 | 0.96821 |

**Table S4: SDMs processing results**. The number of occurrences after cleaning is denominated as Unique occurrences. The table also shows the predictive performance of the models, which were evaluated by the Jaccard Index and AUC. The THR (Jaccard) value corresponds to the threshold values per species after the ensemble and before the overlap of the vegetation cover model.

| **Species** | **Unique ocurrences** | **Jaccard Index** | **AUC** | **THR (Jaccard)** | **True Positive Rate** | **True Negative Rate** |
| --- | --- | --- | --- | --- | --- | --- |
| *Atelocynus microtus* | 281 | 0.931 | 0.988 | 0.535 | 0.987 | 0.981 |
| *Bassaricyon alleni* | 58 | 0.859 | 0.968 | 0.449 | 1 | 0.921 |
| *Cerdocyon thous* | 4051 | 0.969 | 0.997 | 0.699 | 0.991 | 0.992 |
| *Eira barbara* | 2264 | 0.955 | 0.995 | 0.693 | 0.985 | 0.994 |
| *Galictis vittata* | 312 | 0.978 | 0.997 | 0.626 | 0.995 | 0.995 |
| *Herpailurus yagouaroundi* | 2030 | 0.955 | 0.995 | 0.424 | 0.989 | 0.981 |
| *Leopardus pardalis* | 2970 | 0.959 | 0.996 | 0.760 | 0.986 | 0.996 |
| *Leopardus tigrinus* | 413 | 0.970 | 0.996 | 0.453 | 1 | 0.989 |
| *Leopardus wiedii* | 1480 | 0.948 | 0.996 | 0.713 | 0.988 | 0.992 |
| *Mustela africana* | 14 | 0.888 | 0.916 | 0.865 | 1 | 0.833 |
| *Nasua nasua* | 2247 | 0.937 | 0.993 | 0.253 | 0.992 | 0.956 |
| *Panthera onca* | 2492 | 0.957 | 0.996 | 0.729 | 0.985 | 0.994 |
| *Potos flavus* | 1050 | 0.967 | 0.997 | 0.503 | 0.994 | 0.990 |
| *Procyon cancrivorus* | 2285 | 0.961 | 0.996 | 0.634 | 0.994 | 0.987 |
| *Puma concolor* | 4652 | 0.962 | 0.997 | 0.769 | 0.981 | 0.997 |
| *Speothos venaticus* | 434 | 0.969 | 0.997 | 0.639 | 0.988 | 0.994 |

**Table S5. Algorithm performance and threshold.** Performance of the five algorithms used for model building (Maxent - MXS, Support Vector Machine - SPV, Random Forest - RF, General Linear Model and Gaussian Model - GAU). The table contains the Jaccard Index values of each algorithm by species (THR). It also includes the values of True Positive Rate (TPR) and True Negative Rate (TNR) per algorithm and species.

| Species | Algorithm | THR | THR VALUE | TPR | TNR |
| --- | --- | --- | --- | --- | --- |
| *A. microtis* | MXS | Jaccard | 0.598 | 0.774 | 0.995 |
| *A. microtis* | SVM | Jaccard | 0.588 | 0.993 | 0.963 |
| *A. microtis* | RDF | Jaccard | 0.646 | 1 | 1 |
| *A. microtis* | GLM | Jaccard | 0.999 | 1 | 1 |
| *A. microtis* | GAU | Jaccard | 0.668 | 0.981 | 0.969 |
| *B. alleni* | MXS | Jaccard | 0.965 | 0.450 | 0.999 |
| *B. alleni* | SVM | Jaccard | 0.315 | 1 | 0.843 |
| *B. alleni* | RDF | Jaccard | 0.678 | 1 | 1 |
| *B. alleni* | GLM | Jaccard | 0.999 | 1 | 1 |
| *B. alleni* | GAU | Jaccard | 0.578 | 1 | 0.921 |
| *C. thous* | MXS | Jaccard | 0.406 | 0.866 | 0.991 |
| *C. thous* | SVM | Jaccard | 0.699 | 0.992 | 0.987 |
| *C. thous* | RDF | Jaccard | 0.529 | 1 | 0.999 |
| *C. thous* | GLM | Jaccard | 0.576 | 0.991 | 0.979 |
| *C. thous* | GAU | Jaccard | 0.597 | 0.991 | 0.986 |
| *E. barbara* | MXS | Jaccard | 0.488 | 0.850 | 0.994 |
| *E. barbara* | SVM | Jaccard | 0.458 | 0.992 | 0.973 |
| *E. barbara* | RDF | Jaccard | 0.578 | 1 | 0.999 |
| *E. barbara* | GLM | Jaccard | 0.522 | 0.992 | 0.973 |
| *E. barbara* | GAU | Jaccard | 0.572 | 0.992 | 0.973 |
| *G. vittata* | MXS | Jaccard | 0.686 | 0.786 | 0.998 |
| *G. vittata* | SVM | Jaccard | 0.909 | 0.995 | 0.990 |
| *G. vittata* | RDF | Jaccard | 0.738 | 1 | 1 |
| *G. vittata* | GLM | Jaccard | 0.999 | 1 | 1 |
| *G. vittata* | GAU | Jaccard | 0.692 | 0.995 | 0.985 |
| *H. yagouaroundi* | MXS | Jaccard | 0.340 | 0.835 | 0.991 |
| *H. yagouaroundi* | SVM | Jaccard | 0.472 | 0.989 | 0.974 |
| *H. yagouaroundi* | RDF | Jaccard | 0.519 | 0.999 | 0.997 |
| *H. yagouaroundi* | GLM | Jaccard | 0.466 | 0.987 | 0.968 |
| *H. yagouaroundi* | GAU | Jaccard | 0.602 | 0.984 | 0.979 |
| *L. pardalis* | MXS | Jaccard | 0.357 | 0.829 | 0.990 |
| *L. pardalis* | SVM | Jaccard | 0.573 | 0.991 | 0.970 |
| *L. pardalis* | RDF | Jaccard | 0.560 | 1 | 1 |
| *L. pardalis* | GLM | Jaccard | 0.483 | 0.989 | 0.969 |
| *L. pardalis* | GAU | Jaccard | 0.595 | 0.990 | 0.973 |
| *L. tigrinus* | MXS | Jaccard | 0.647 | 0.725 | 0.997 |
| *L. tigrinus* | SVM | Jaccard | 0.554 | 1 | 0.983 |
| *L. tigrinus* | RDF | Jaccard | 0.633 | 1 | 1 |
| *L. tigrinus* | GLM | Jaccard | 0.999 | 1 | 1 |
| *L. tigrinus* | GAU | Jaccard | 0.458 | 1 | 0.979 |
| *L. wiedii* | MXS | Jaccard | 0.462 | 0.778 | 0.993 |
| *L. wiedii* | SVM | Jaccard | 0.386 | 0.997 | 0.964 |
| *L. wiedii* | RDF | Jaccard | 0.566 | 1 | 0.998 |
| *L. wiedii* | GLM | Jaccard | 0.677 | 0.987 | 0.982 |
| *L. wiedii* | GAU | Jaccard | 0.631 | 0.988 | 0.977 |
| *M. africana* | MXS | Jaccard | 0.857 | 0.333 | 0.988 |
| *M. africana* | SVM | Jaccard | 0.750 | 1 | 1 |
| *M. africana* | RDF | Jaccard | 0.865 | 1 | 1 |
| *M. africana* | GLM | Jaccard | 0 | 0 | 0 |
| *M. africana* | GAU | Jaccard | 0.647 | 1 | 1 |
| *N. nasua* | MXS | Jaccard | 0.709 | 0.768 | 0.996 |
| *N. nasua* | SVM | Jaccard | 0.154 | 0.998 | 0.952 |
| *N. nasua* | RDF | Jaccard | 0.526 | 1 | 0.999 |
| *N. nasua* | GLM | Jaccard | 0.426 | 0.990 | 0.958 |
| *N. nasua* | GAU | Jaccard | 0.461 | 0.994 | 0.953 |
| *P. onca* | MXS | Jaccard | 0.371 | 0.838 | 0.988 |
| *P. onca* | SVM | Jaccard | 0.560 | 0.989 | 0.978 |
| *P. onca* | RDF | Jaccard | 0.485 | 0.998 | 0.999 |
| *P. onca* | GLM | Jaccard | 0.585 | 0.987 | 0.975 |
| *P. onca* | GAU | Jaccard | 0.590 | 0.989 | 0.979 |
| *P. flavus* | MXS | Jaccard | 0.245 | 0.895 | 0.996 |
| *P. flavus* | SVM | Jaccard | 0.623 | 0.993 | 0.979 |
| *P. flavus* | RDF | Jaccard | 0.590 | 1 | 1 |
| *P. flavus* | GLM | Jaccard | 0.498 | 0.998 | 0.994 |
| *P. flavus* | GAU | Jaccard | 0.692 | 0.987 | 0.987 |
| *P. cancrivorus* | MXS | Jaccard | 0.481 | 0.793 | 0.993 |
| *P. cancrivorus* | SVM | Jaccard | 0.268 | 0.997 | 0.969 |
| *P. cancrivorus* | RDF | Jaccard | 0.528 | 0.999 | 0.999 |
| *P. cancrivorus* | GLM | Jaccard | 0.555 | 0.988 | 0.976 |
| *P. cancrivorus* | GAU | Jaccard | 0.549 | 0.996 | 0.972 |
| *P. concolor* | MXS | Jaccard | 0.561 | 0.798 | 0.984 |
| *P. concolor* | SVM | Jaccard | 0.501 | 0.990 | 0.977 |
| *P. concolor* | RDF | Jaccard | 0.473 | 0.999 | 0.998 |
| *P. concolor* | GLM | Jaccard | 0.357 | 0.993 | 0.954 |
| *P. concolor* | GAU | Jaccard | 0.635 | 0.985 | 0.973 |
| *S. venaticus* | MXS | Jaccard | 0.574 | 0.764 | 0.996 |
| *S. venaticus* | SVM | Jaccard | 0.904 | 0.982 | 0.994 |
| *S. venaticus* | RDF | Jaccard | 0.525 | 1 | 1 |
| *S. venaticus* | GLM | Jaccard | 0.999 | 1 | 1 |
| *S. venaticus* | GAU | Jaccard | 0.775 | 0.982 | 0.991 |

**Figure S1**

**
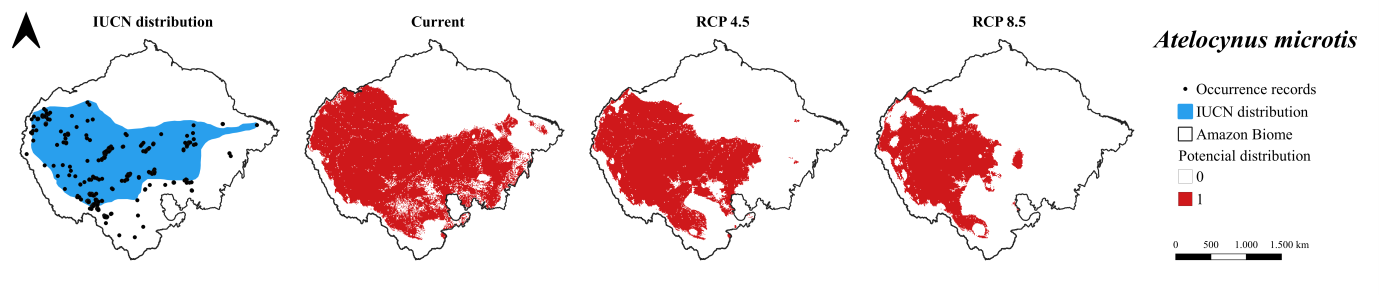
**

**
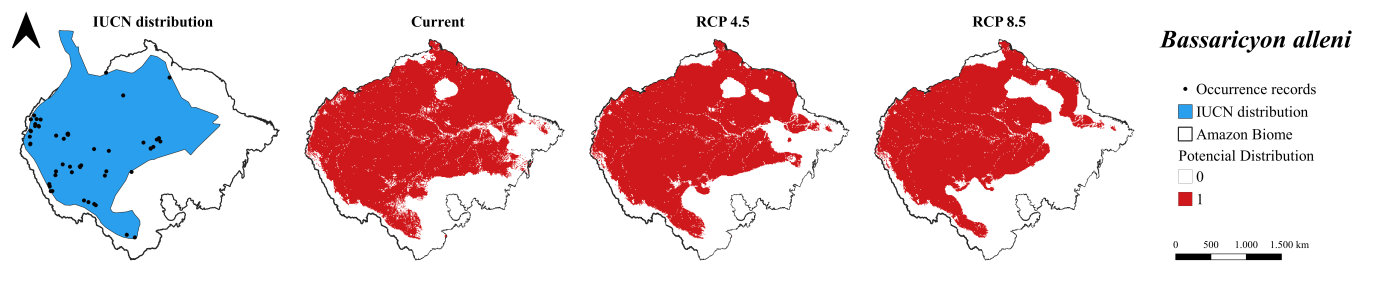
**

**
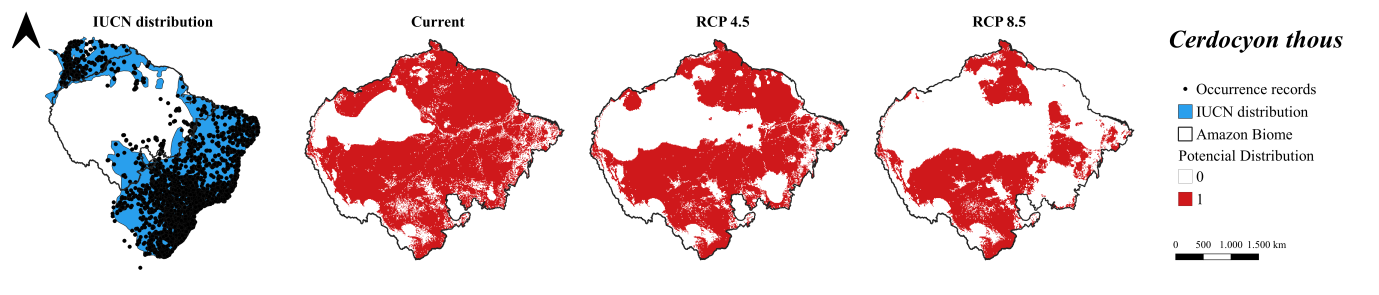
**

**
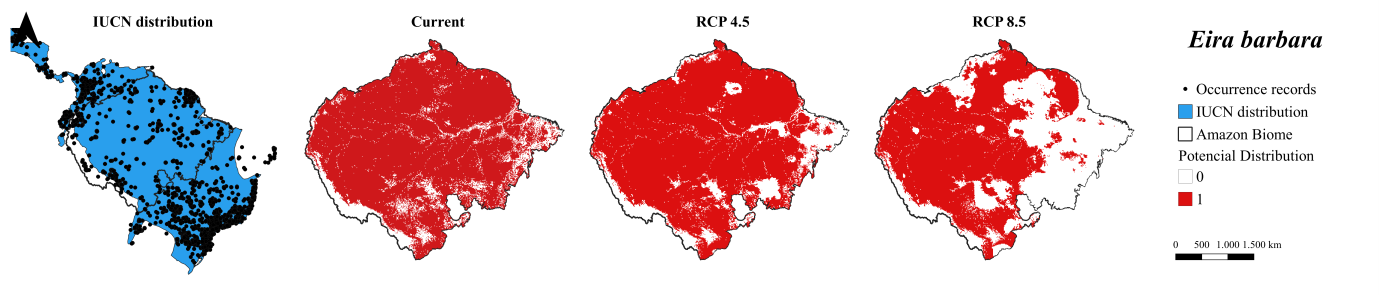
**

**
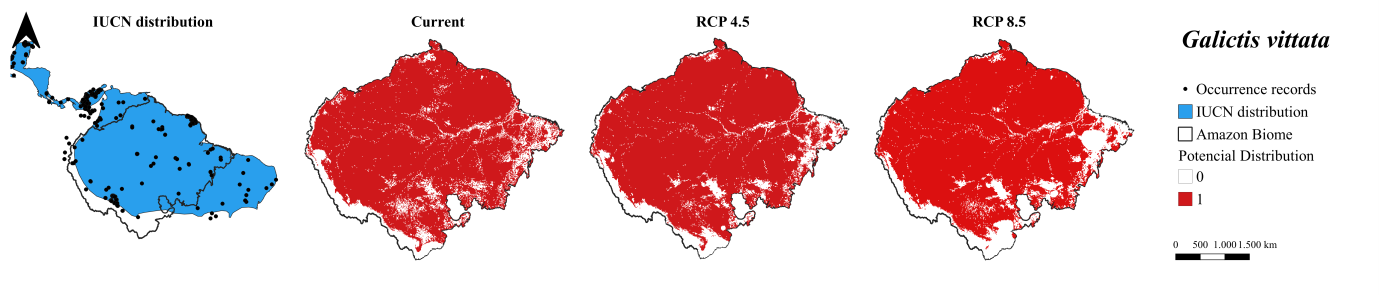
**

**
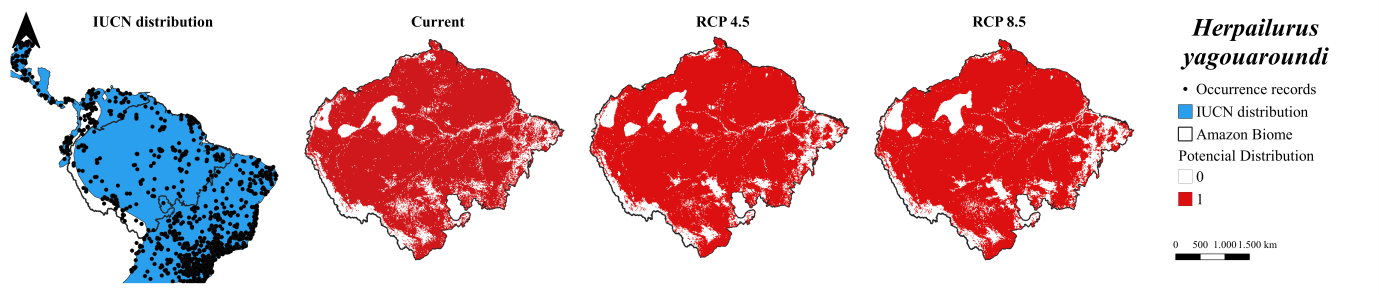
**

**
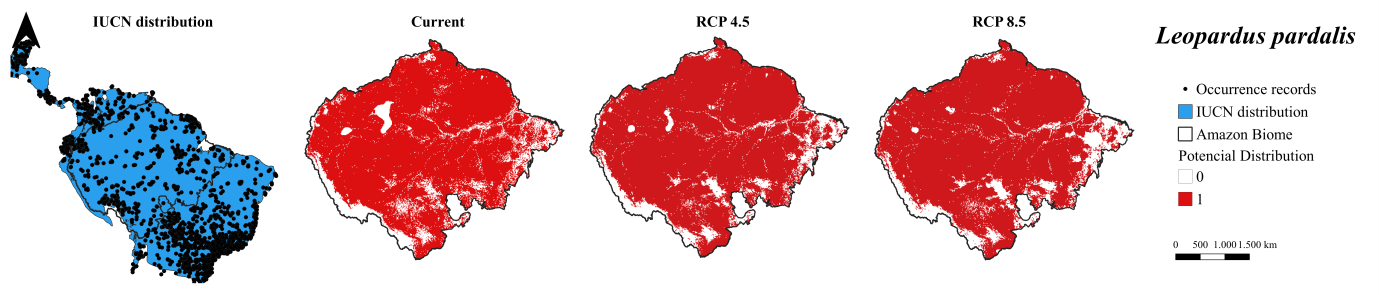
**

**
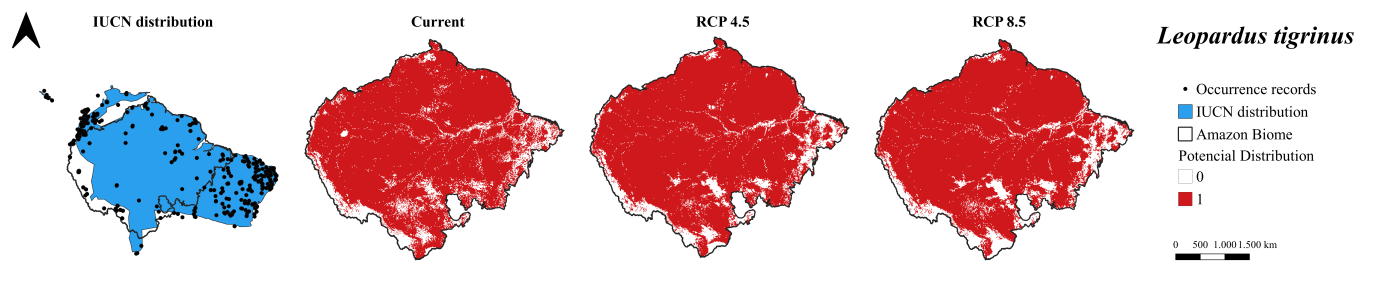
**

**
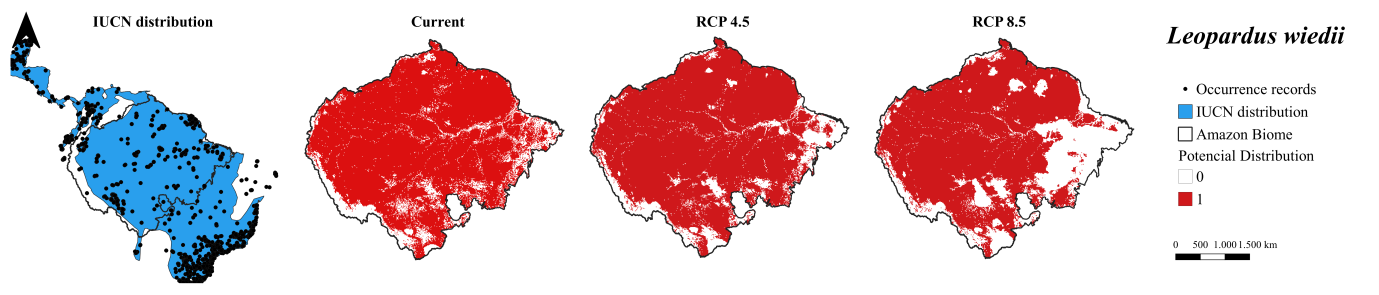
**

**
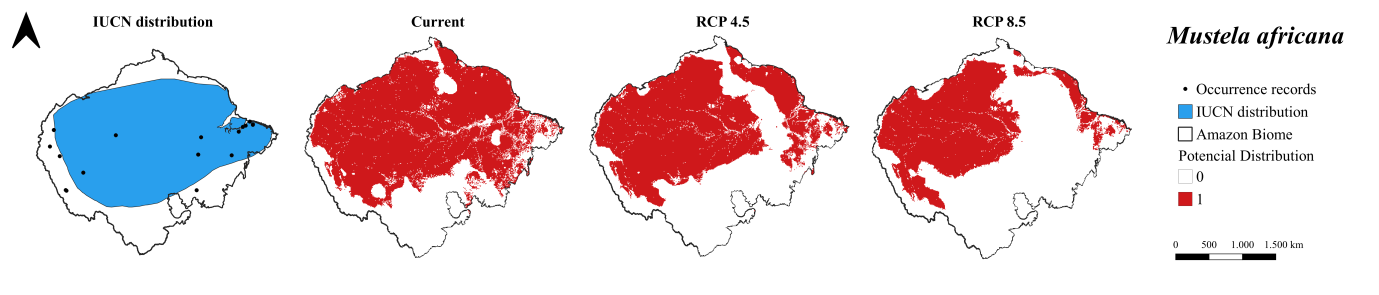
**

**
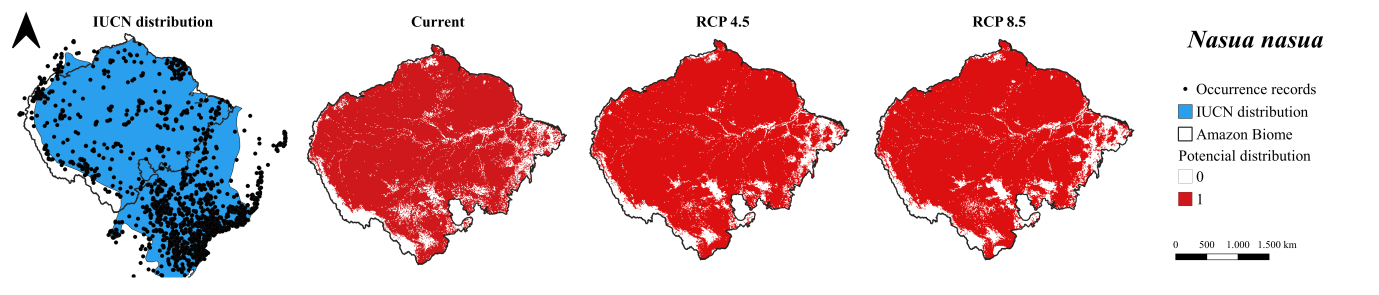
**

**
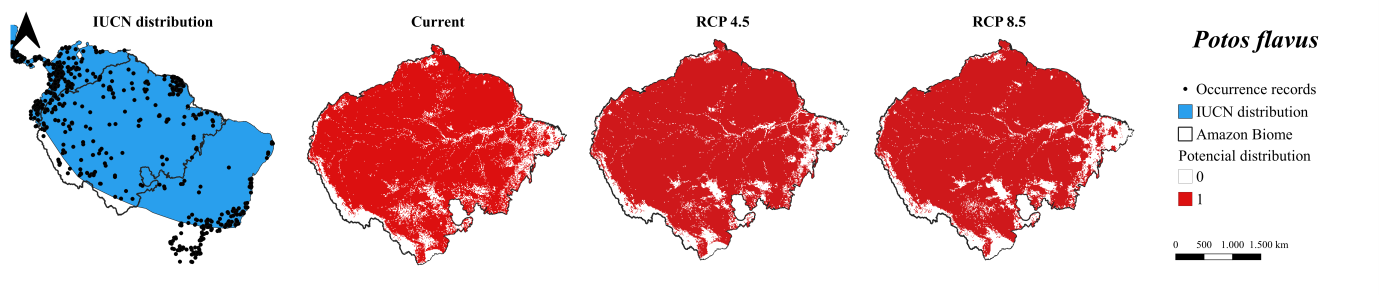
**

**
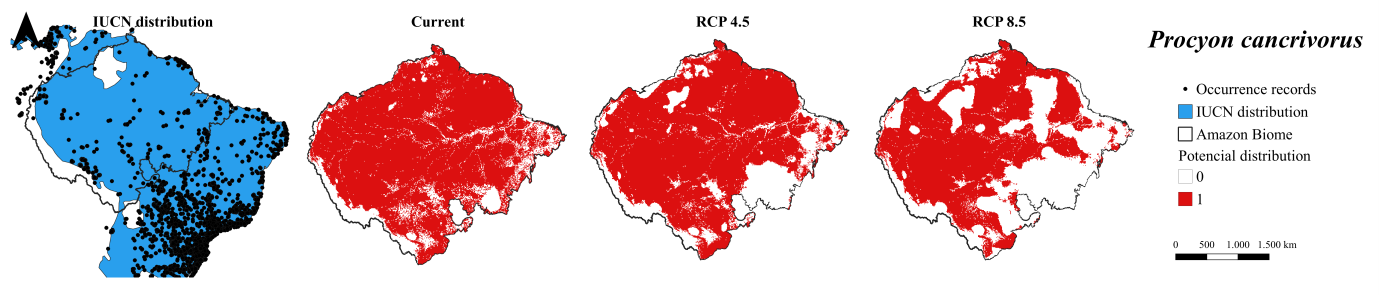
**

**
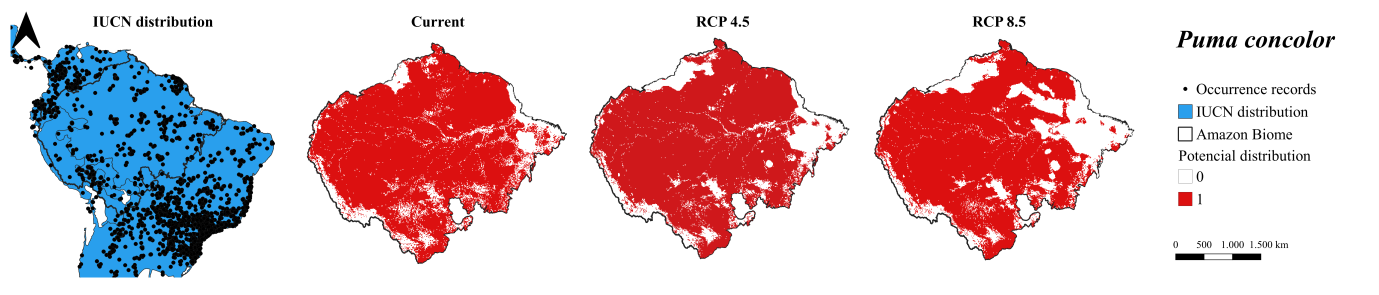
**

**
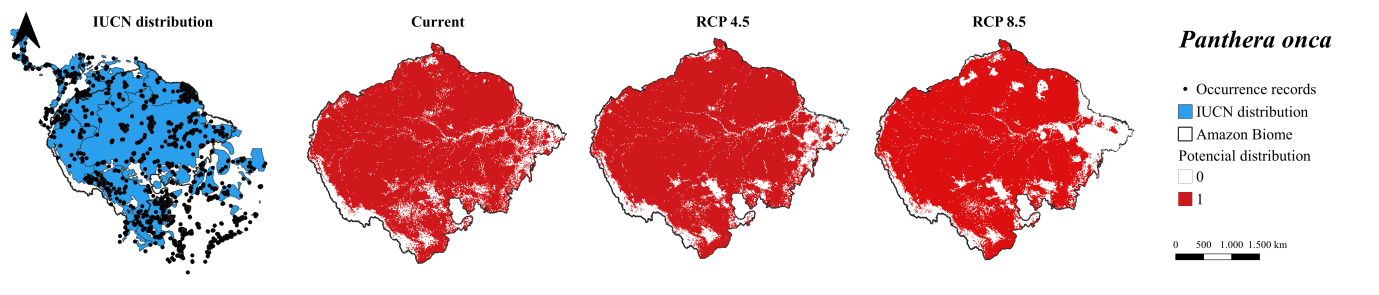
**

**
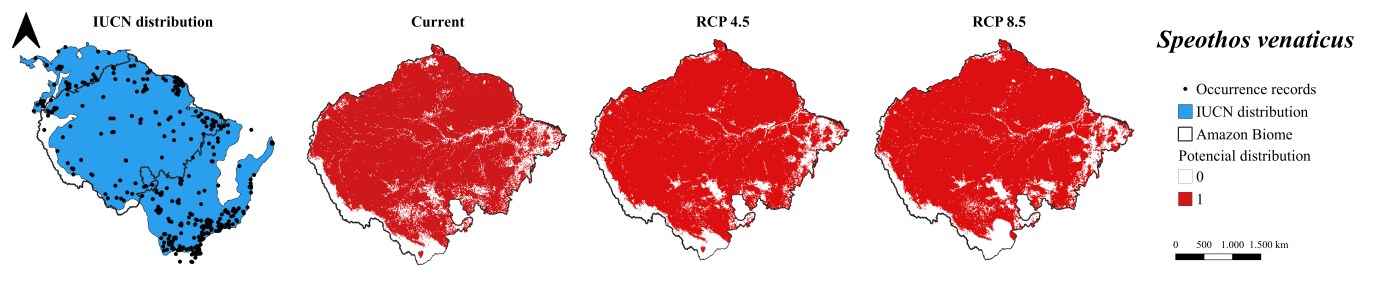
**

**Figure S1: Suitability models for carnivorous mammals of the Amazon Biome on all climate scenarios.** Black lines and white area: Amazon Biome. From left to right: geographical distribution of carnivore species according to IUCN; potential distribution for the current-time, potential distribution in RCP 4.5 and potential distribution in RCP 8.5. All the models presented above are the result of overlapping the vegetation cover models to the consensus SDMs of each species.

**Table S6: Measurement of the spatial dynamics of carnivores**. Area represents the total suitability area of the species. Negative values of Lost/Gain Area represent the loss of suitable area of the species, and positive values represent gain of suitable area (km^2^ and percentage). The species are stable if they present negative and positive values below 5%.

| Area (km^2^) | Current | RCP 45 | RCP 85 |  | Lost/Gain Area | | | |
| --- | --- | --- | --- | --- | --- | --- | --- | --- |
|  |  | Total | Total |  | RCP 45 (km^2^) | RCP 85 (km^2^) | RCP 45 (%) | RCP 85 (%) |
| *A. microtis* | 3978658.2 | 2943452.82 | 2019241.89 |  | -1035205.38 | -1959416.31 | -26.02 | -49.25 |
| *B. alleni* | 5186569.23 | 4927296.48 | 4306156.11 |  | -259272.75 | -880413.12 | -5.00 | -16.97 |
| *C. thous* | 5158284.93 | 4253958.72 | 2790717.6 |  | -904326.21 | -2367567.33 | -17.53 | -45.90 |
| *E. barbara* | 6327369.33 | 6198804.33 | 4470205.05 |  | -128565 | -1857164.28 | -2.03 | -29.35 |
| *G. vittata* | 6215946.33 | 6330712.02 | 6110608.74 |  | 114765.69 | -105337.59 | 1.85 | -1.69 |
| *H. yagouaroundi* | 6054554.4 | 6178919.61 | 6101352.06 |  | 124365.21 | 46797.66 | 2.05 | 0.77 |
| *L. pardalis* | 6226574.37 | 6407851.02 | 6308427.42 |  | 181276.65 | 81853.05 | 2.91 | 1.31 |
| *L. tigrinus* | 6346139.82 | 6453791.58 | 6357024.99 |  | 107651.76 | 10885.17 | 1.70 | 0.17 |
| *L. wiedii* | 6279543.15 | 6276714.72 | 5408815.26 |  | -2828.43 | -870727.89 | -0.05 | -13.87 |
| *M. africana* | 4978722.48 | 4161734.76 | 2998821.48 |  | -816987.72 | -1979901 | -16.41 | -39.77 |
| *N. nasua* | 6342025.74 | 6461419.77 | 6370310.04 |  | 119394.03 | 28284.3 | 1.88 | 0.45 |
| *P. flavus* | 6311170.14 | 6413079.33 | 6295313.79 |  | 101909.19 | -15856.35 | 1.61 | -0.25 |
| *P. cancrivorus* | 6174462.69 | 5711542.98 | 4391608.98 |  | -462919.71 | -1782853.71 | -7.50 | -28.87 |
| *P. onça* | 6332683.35 | 6422764.56 | 6056440.02 |  | 90081.21 | -276243.33 | 1.42 | -4.36 |
| *P. concolor* | 6143092.83 | 6015042.09 | 5474983.38 |  | -128050.74 | -668109.45 | -2.08 | -10.88 |
| *S. venaticus* | 6245259.15 | 6358824.9 | 6216889.14 |  | 113565.75 | -28370.01 | 1.82 | -0.45 |

**Figure S2**

**
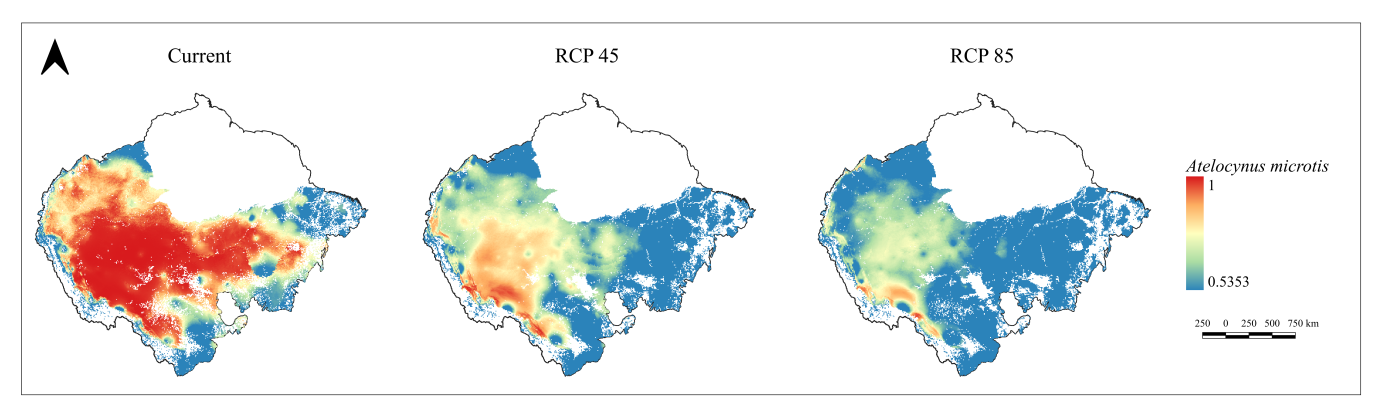
**

**
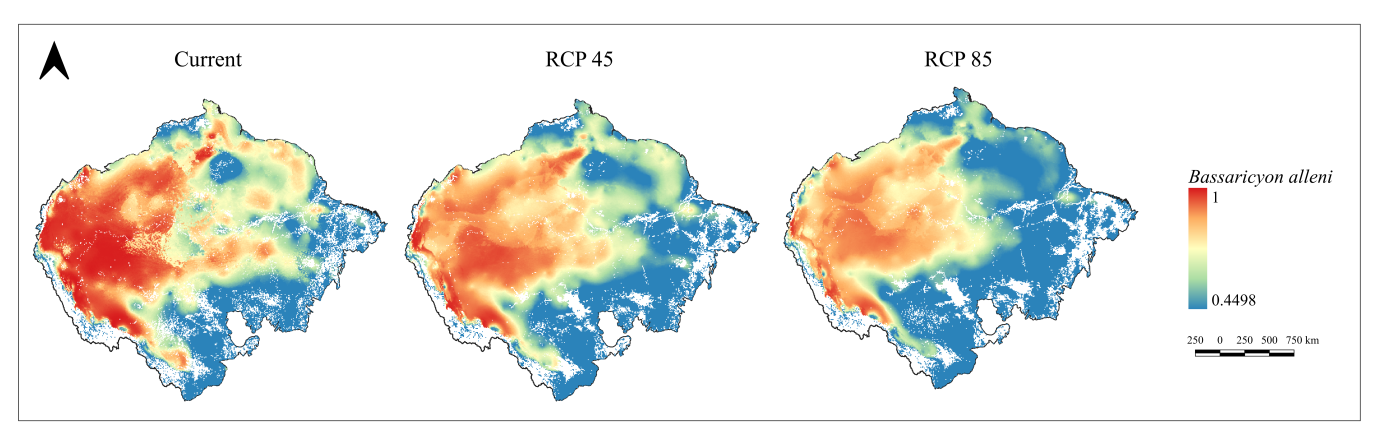
**

**
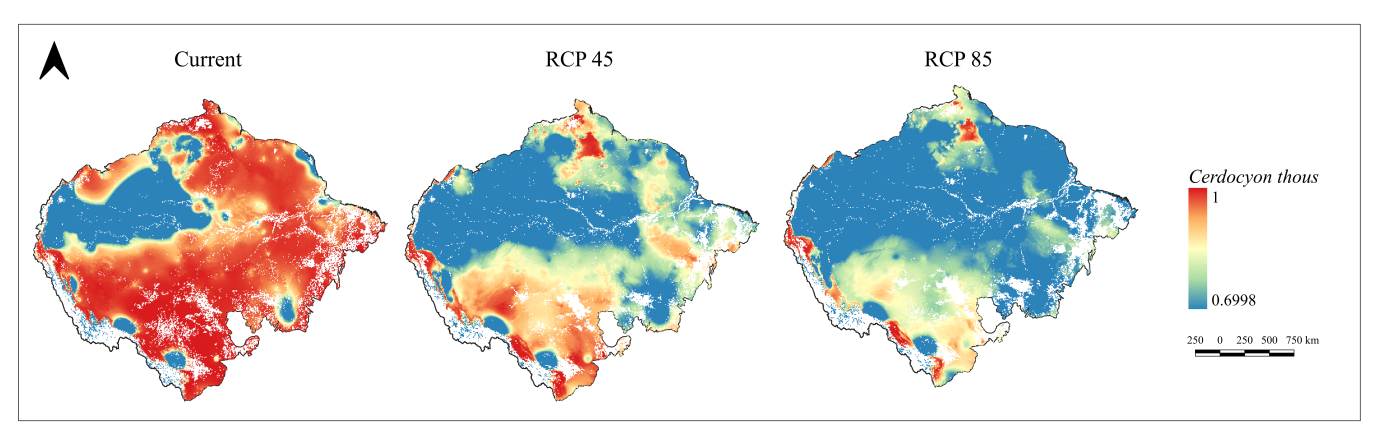
**

**
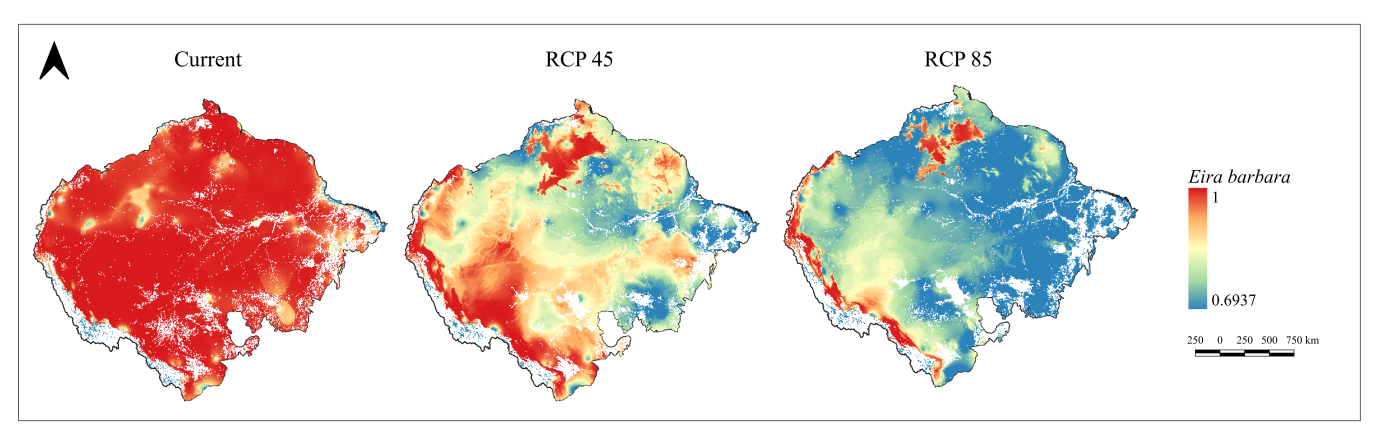
**

**
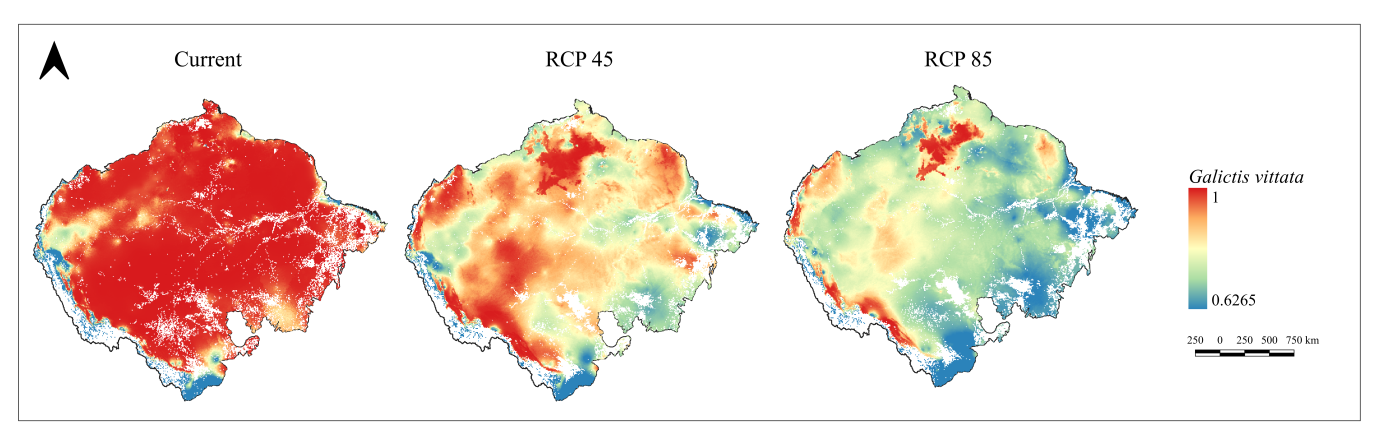
**

**
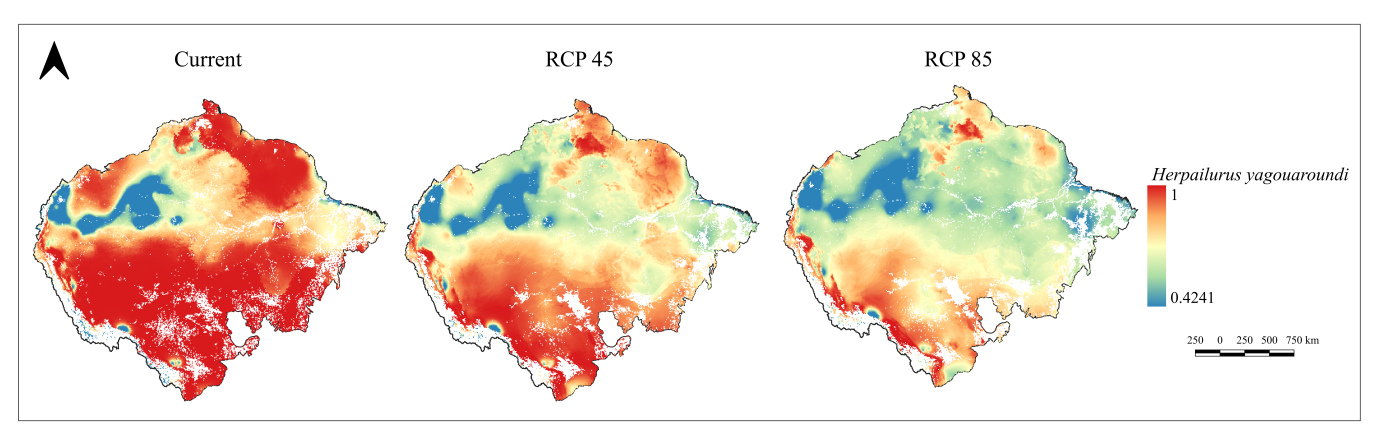
**

**
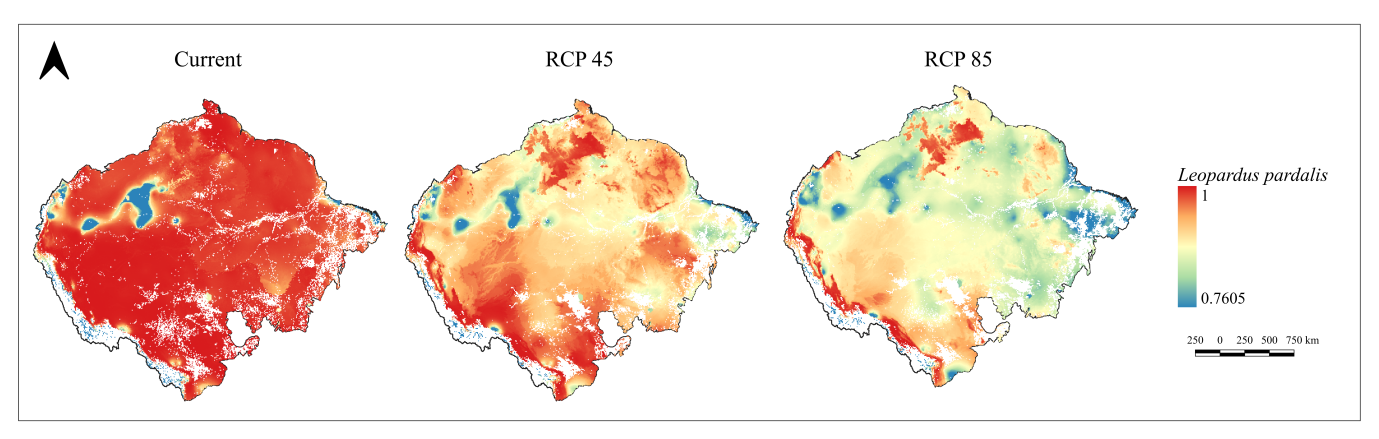
**

**
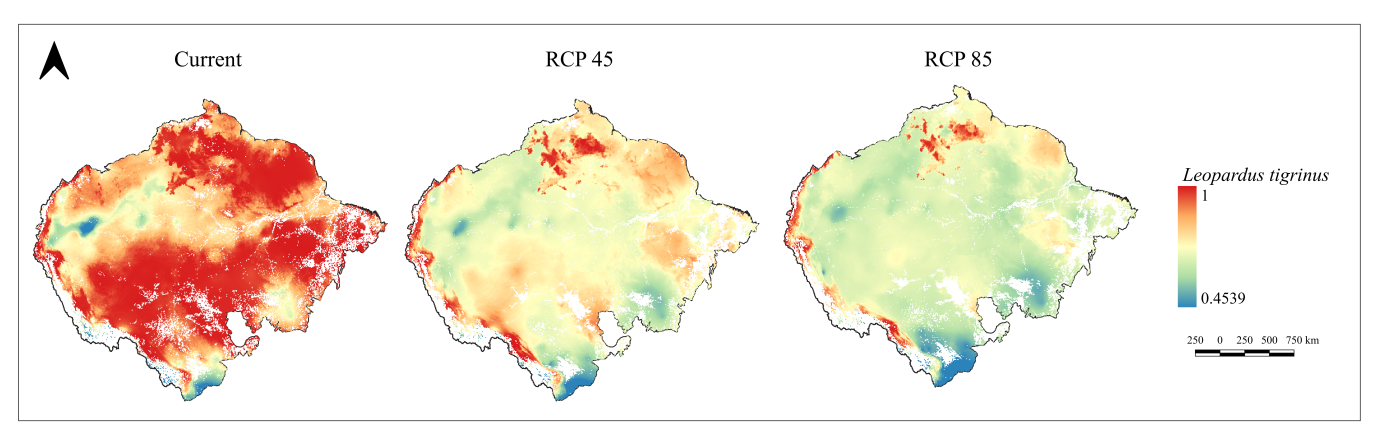
**

**
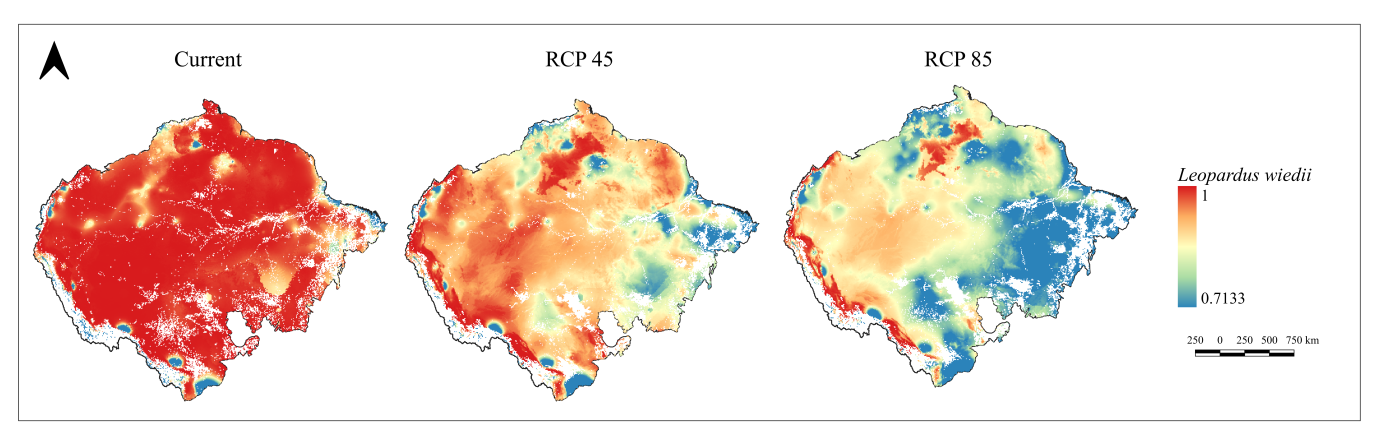
**

**
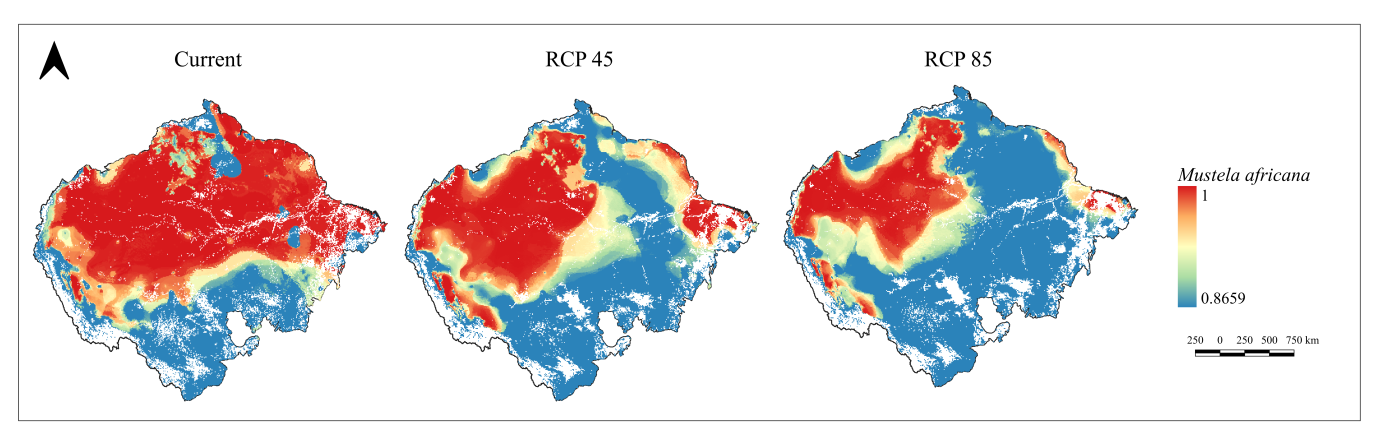
**

**
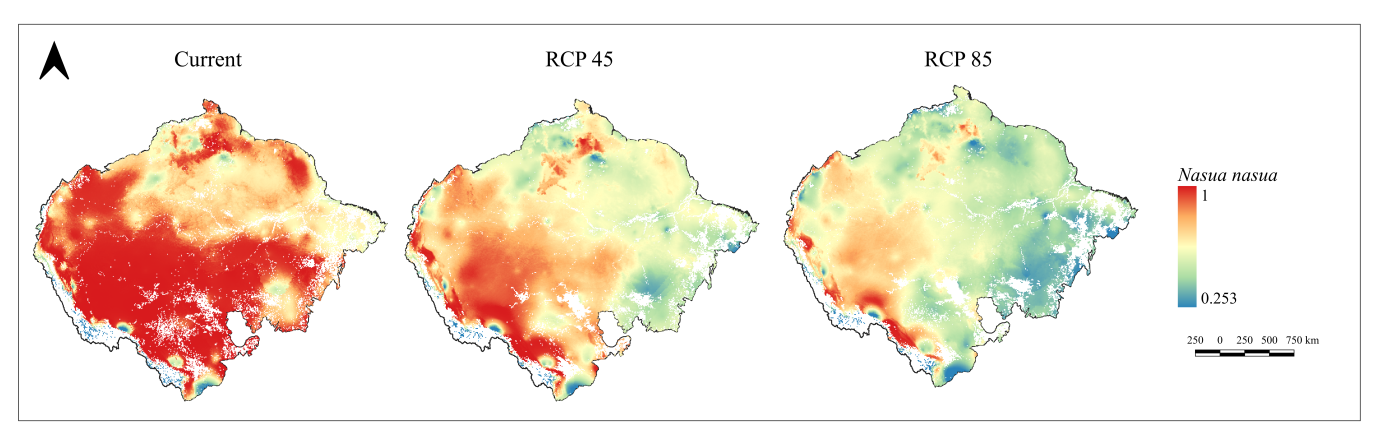
**

**
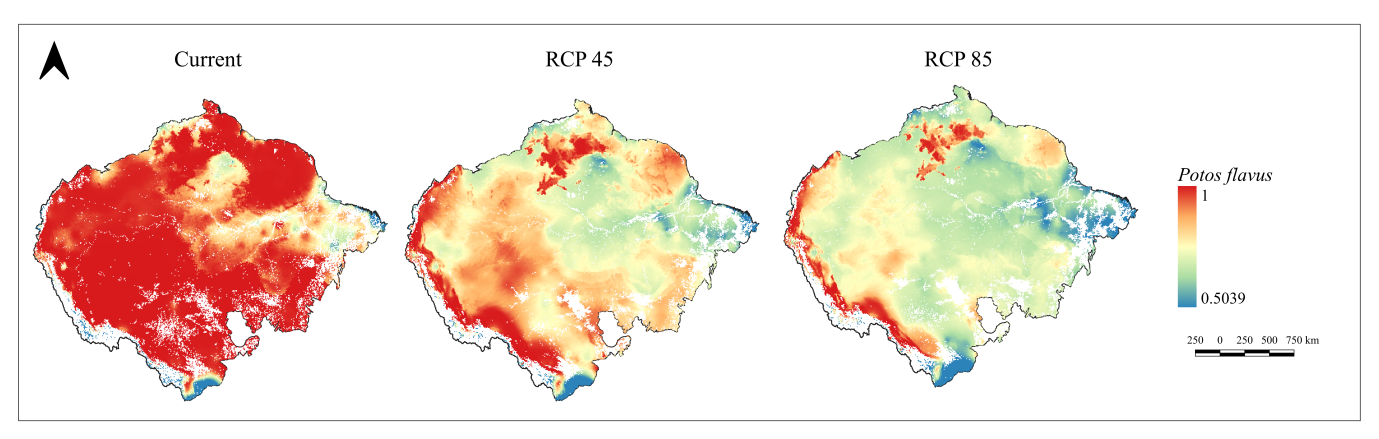
**

**
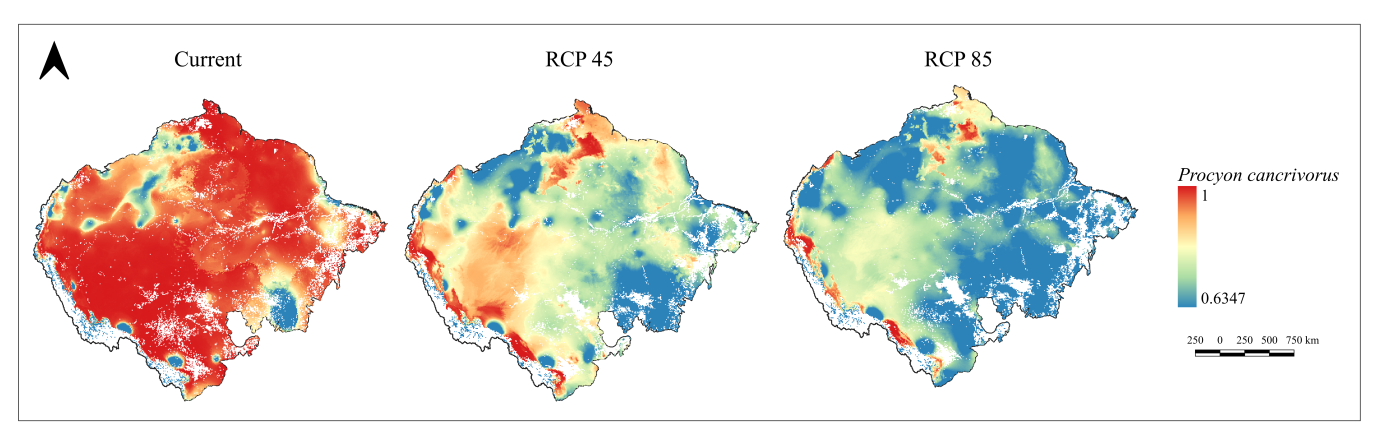
**

**
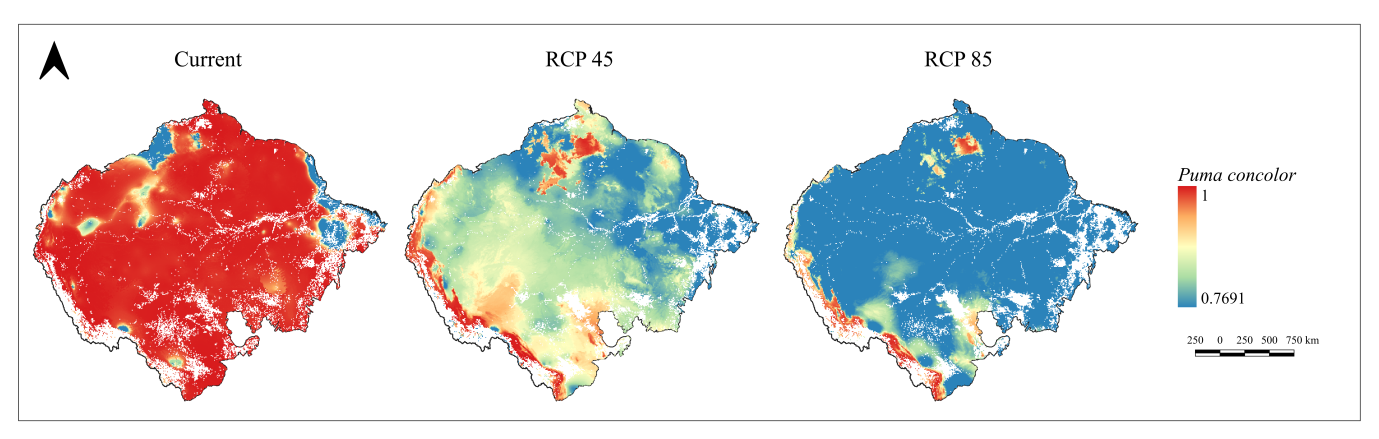
**

**
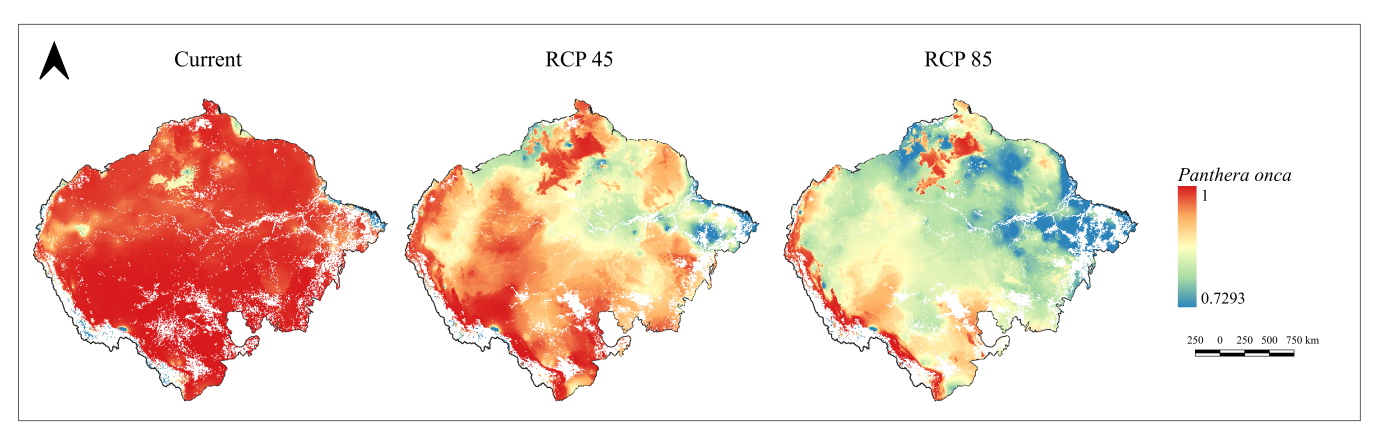
**

**
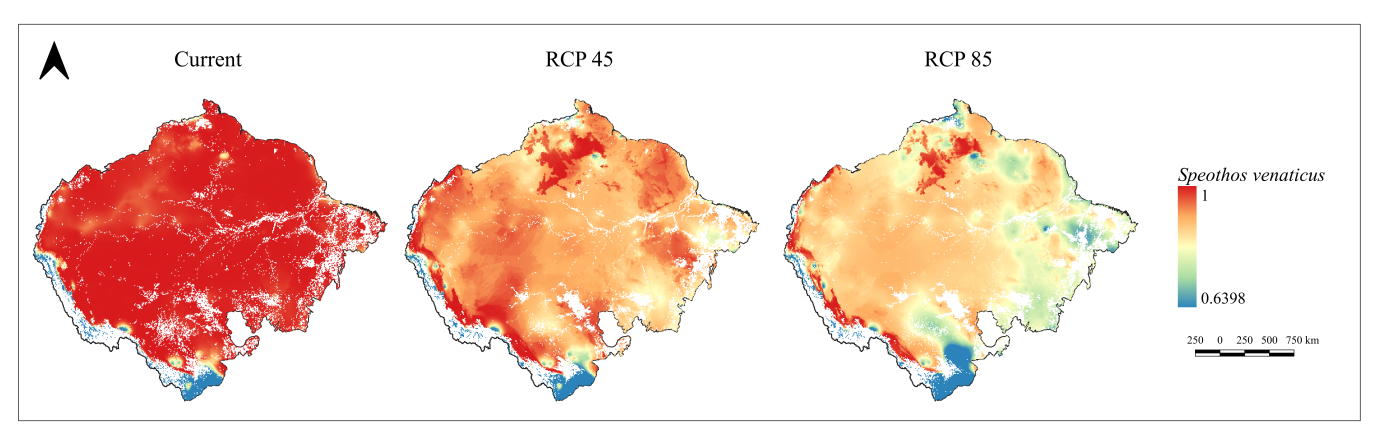
**

**Figure S2: Climatic suitability models for carnivorous mammals of the Amazon Biome on all climate scenarios.** Black line and white area: Amazon Biome. From left to right: climatic suitability for the current-time, climatic suitability in RCP 4.5 and climatic suitability in RCP 8.5. The minimum climate suitability values for each species correspond to the THR (Jaccard) cited in Table S4. All the models presented above are the result of overlapping the vegetation cover models to the consensus SDMs of each species.

**Table S7: Results of GAP analysis.** Target for protected rating is 0.1 (10%). All species are protected. IPA: Integral Protected Areas; SUA: Sustainable Use Areas; and IL: Indigenous Lands.

| **Current** | **IPA** | **IPA+SUA** | **IPA+SUA+IL** |
| --- | --- | --- | --- |
| Species | Target achieved | Target achieved | Target achieved |
| *A. microtis* | 1.315 | 3.299 | 6.648 |
| *B. alleni* | 1.787 | 4.133 | 7.043 |
| *C. thous* | 1.531 | 3.700 | 6.364 |
| *E. barbara* | 1.572 | 3.730 | 6.625 |
| *G. vittata* | 1.587 | 3.745 | 6.624 |
| *H. yagouaroundi* | 1.585 | 3.782 | 6.558 |
| *L. pardalis* | 1.580 | 3.753 | 6.605 |
| *L. tigrinus* | 1.569 | 3.728 | 6.620 |
| *L. wiedii* | 1.579 | 3.720 | 6.612 |
| *M. africana* | 1.697 | 3.958 | 6.831 |
| *N. nasua* | 1.570 | 3.738 | 6.631 |
| *P. flavus* | 1.577 | 3.729 | 6.615 |
| *P. cancrivorus* | 1.589 | 3.777 | 6.650 |
| *P. onca* | 1.571 | 3.720 | 6.621 |
| *P. concolor* | 1.572 | 3.681 | 6.597 |
| *S. venaticus* | 1.590 | 3.748 | 6.629 |
|  |  |  |  |
| **RCP 4.5** | **IPA** | **IPA+SUA** | **IPA+SUA+IL** |
| Species | Target achieved | Target achieved | Target achieved |
| *A. microtis* | 1.392 | 3.385 | 6.900 |
| *B. alleni* | 1.791 | 4.061 | 7.007 |
| *C. thous* | 1.533 | 3.620 | 6.298 |
| *E. barbara* | 1.603 | 3.630 | 6.693 |
| *G. vittata* | 1.582 | 3.585 | 6.625 |
| *H. yagouaroundi* | 1.574 | 3.632 | 6.560 |
| *L. pardalis* | 1.560 | 3.580 | 6.621 |
| *L. tigrinus* | 1.559 | 3.578 | 6.614 |
| *L. wiedii* | 1.589 | 3.589 | 6.637 |
| *M. africana* | 1.710 | 3.793 | 6.871 |
| *N. nasua* | 1.558 | 3.581 | 6.628 |
| *P. flavus* | 1.568 | 3.570 | 6.602 |
| *P. cancrivorus* | 1.642 | 3.790 | 6.722 |
| *P. onca* | 1.565 | 3.568 | 6.627 |
| *P. concolor* | 1.562 | 3.572 | 6.615 |
| *S. venaticus* | 1.579 | 3.592 | 6.625 |
|  |  |  |  |
| **RCP 8.5** | **IPA** | **IPA+SUA** | **IPA+SUA+IL** |
| Species | Target achieved | Target achieved | Target achieved |
| *A. microtis* | 1.307 | 3.364 | 6.840 |
| *B. alleni* | 1.780 | 4.072 | 7.069 |
| *C. thous* | 1.509 | 3.780 | 6.715 |
| *E. barbara* | 1.778 | 3.995 | 7.021 |
| *G. vittata* | 1.635 | 3.748 | 6.749 |
| *H. yagouaroundi* | 1.593 | 3.738 | 6.601 |
| *L. pardalis* | 1.592 | 3.705 | 6.689 |
| *L. tigrinus* | 1.583 | 3.682 | 6.654 |
| *L. wiedii* | 1.680 | 3.847 | 6.784 |
| *M. africana* | 1.730 | 3.829 | 7.127 |
| *N. nasua* | 1.580 | 3.694 | 6.670 |
| *P. flavus* | 1.595 | 3.689 | 6.664 |
| *P. cancrivorus* | 1.602 | 3.873 | 6.805 |
| *P. onca* | 1.602 | 3.711 | 6.751 |
| *P. concolor* | 1.520 | 3.591 | 6.653 |
| *S. venaticus* | 1.613 | 3.724 | 6.692 |
|  |  |  |  |

**Figure S3: Results of Null Model.** In all climate scenarios, PAs did not reach different values expected by chance (p < 0.05). Minimum and maximums values of p for all PAs. The minimum p-value was 0.05 and the maximum p-value was 1.
